# Novel Plasma Proteomic Markers and Risk of Venous Thromboembolism

**DOI:** 10.1161/CIRCULATIONAHA.125.074493

**Published:** 2026-02-16

**Authors:** Weihong Tang, Aixin Li, Thomas R. Austin, Sigrid K. Brækkan, Therese H. Nøst, Xumin Li, Rajat Deo, Ruth Dubin, Peter Ganz, Weihua Guan, Rui Cao, John-Bjarne Hansen, Kristian Hveem, Ron C. Hoogeveen, Christian Jonasson, Jerome I. Rotter, Kunihiro Matsushita, Guning Liu, James S. Pankow, Nathan Pankratz, Bruce M. Psaty, Kent D. Taylor, Florian Thibord, Eric Boerwinkle, Nicholas L. Smith, Mary Cushman, Aaron R. Folsom

**Affiliations:** Division of Epidemiology & Community Health, School of Public Health, University of Minnesota, Minneapolis, MN (W.T., A.L., J.S.P., A.R.F.).; Cardiovascular Health Research Unit, Department of Epidemiology, University of Washington, Seattle, WA (T.R.A.).; Thrombosis Research Center (TREC), Division of Internal Medicine, University Hospital of North Norway, Tromsø, Norway (S.K.B., J.-B.H.).; Thrombosis Research group (TREC), Department of Clinical Medicine, UiT The Arctic University of Norway, Tromsø, Norway (S.K.B., J.-B.H.).; HUNT Research Center, Norwegian University of Science and Technology, Levanger, Norway (T.H.N., K.H., C.J.).; Levanger Hospital, Nord-Trøndelag Hospital Trust, Levanger, Norway (T.H.N.).; HUNT Center for Molecular and Clinical Epidemiology, Norwegian University of Science and Technology, Trondheim, Norway (T.H.N., K.H.).; Department of Community Medicine, UiT The Arctic University of Norway, Tromsø, Norway (T.H.N.).; Department of Epidemiology, University of Washington, Seattle, WA (X.L., N.L.S.).; Division of Cardiovascular Medicine, Perelman School of Medicine at the University of Pennsylvania, Philadelphia, PA (R. Deo).; Division of Nephrology, University of Texas Southwestern Medical Center, Dallas, TX (R. Dubin).; Division of Cardiology, University of California, San Francisco, San Francisco, CA (P.G.).; Division of Biostatistics & Health Data Science, University of Minnesota, Minneapolis, MN (W.G., R.C.).; Division of Cardiovascular Research, Department of Medicine, Baylor College of Medicine, Houston, TX (R.C.H.).; The Institute for Translational Genomics and Population Sciences, Department of Pediatrics, The Lundquist Institute for Biomedical Innovation at Harbor-UCLA Medical Center, Torrance, CA (J.I.R., K.D.T.).; Department of Epidemiology, Johns Hopkins Bloomberg School of Public Health, Baltimore, MD (K.M.).; Department of Epidemiology, Human Genetics and Environmental Sciences, The University of Texas Health Science Center, School of Public Health, Houston, TX (G.L., E.B.).; Department of Laboratory Medicine and Pathology, University of Minnesota Medical School, Minneapolis, MN (N.P.).; Cardiovascular Health Research Unit, Departments of Medicine, Epidemiology, and Health Systems and Population Health, University of Washington, Seattle, WA (B.M.P.).; INSERM UMR 1219, Bordeaux Population Health Research Center, Bordeaux, France (F.T.).; Human Genome Sequencing Center, Baylor College of Medicine, Houston, TX (E.B.).; Kaiser Permanente Washington Health Research Institute, Kaiser Permanente Washington, Seattle, WA (N.L.S.).; Seattle Epidemiologic Research and Information Center, Department of Veterans Affairs Office of Research and Development, Seattle, WA (N.L.S.).; Department of Pathology and Laboratory Medicine, Larner College of Medicine, University of Vermont, Burlington, VT (M.C.).; Department of Medicine, Larner College of Medicine, University of Vermont, Burlington, VT (M.C.).

**Keywords:** biomarkers, etiology, proteomics, risk factors, venous thromboembolism

## Abstract

**BACKGROUND:**

Venous thromboembolism (VTE) is a leading cardiovascular disease, yet its etiology is incompletely understood. This study used large-scale, high-throughput aptamer-based proteomics to identify new circulating protein biomarkers and biological pathways for incident VTE.

**METHODS:**

We included 4 longitudinal cohorts (the ARIC study [Atherosclerosis Risk in Communities], CHS [Cardiovascular Health Study], MESA [Multi-Ethnic Study of Atherosclerosis], and the HUNT study [Trøndelag Health]) that identified 1371 incident noncancer VTEs among 20 737 participants followed for a maximum of 10 to 29 years. We used the SomaScan to measure baseline plasma levels of ≈5000 to 7000 proteins and examined the prospective relationships between the protein biomarkers and noncancer VTE. We then conducted an external replication of top VTE proteins in 783 incident noncancer VTEs among 39 097 participants in the UKB study (UK Biobank) based on the Olink proteomics platform. We used Cox proportional hazards regression to estimate the association between each protein biomarker and VTE risk. Mendelian randomization (MR) analysis was used to assess the possible causal associations between identified proteins and VTE risk.

**RESULTS:**

There were 23 proteins that exceeded a false discovery rate–adjusted *P*<0.05 (unadjusted *P*<6.5×10^-4^) in the discovery meta-analysis of ARIC, CHS, and MESA and were replicated in HUNT at (unadjusted) *P*<0.05. Of these, 15 are new to VTE, and 3 of the 15 (transgelin, sushi, von Willebrand factor type A, EGF and pentraxin domain-containing protein 1, and TIMP4 [metalloproteinase inhibitor 4]) exceeded the Bonferroni corrected significance threshold in HUNT. Sixteen of the 23 top VTE proteins were available on the UKB Olink panel, of which 11 were replicated in the UKB study after Bonferroni correction. MR analysis of the 15 new proteins provided significant evidence for a possible causal role of TIMD4 (T-cell immunoglobulin and mucin domain-containing protein 4) (Bonferroni-corrected *P*<0.05) and suggestive evidence for TIMP4 and CST3 (cystatin-c) (unadjusted *P*<0.05) in VTE risk. The direction of association from the MR analyses was opposite of that from the VTE proteomics analysis for TIMP4 and TIMD4 but was consistent for CST3.

**CONCLUSIONS:**

We identified several novel plasma proteins for VTE that reflect biological processes outside established VTE pathophysiology, including extracellular matrix regulation, immunity, immune–vascular endothelium interactions, and vascular senescence. Results may provide new modifiable targets to improve VTE risk stratification, prevention, or treatment.

**Clinical Perspective**

**What Is New?**

- Because the causes of unprovoked venous thromboembolism (VTE) remain incompletely understood, we tested associations of ≈5000 plasma proteins with risk of future noncancer-related VTE in 5 population-based cohorts.
- We identified 23 proteomic signatures, 15 of which have not been previously described.
- These signatures reflect both established biological processes implicated in VTE and novel pathways beyond traditional VTE pathophysiology, including extracellular matrix regulation, immunity, vascular senescence, and fibrosis.

**What Are the Clinical Implications?**

- These discoveries suggest previously unrecognized pathways involved in the etiology of unprovoked VTE and identify plasma protein markers that may serve as modifiable targets for intervention or be used to improve risk stratification, prevention, and treatment.
- Our findings also suggest that future larger-scale multiplex proteomics studies could uncover additional risk biomarkers and therapeutic targets for prevention of unprovoked VTE.

Venous thromboembolism (VTE), encompassing deep venous thrombosis (DVT) and pulmonary embolism (PE), is a common condition and the third most common life-threatening cardiovascular disease (CVD).^1^ Its etiology is incompletely understood. Whereas hemostasis factors, including higher levels of FVIII (coagulation factor VIII), VWF (von Willebrand factor), and FXI (coagulation factor XI), are considered causal risk factors for VTE,^2–5^ some nonhemostatic pathways, such as the inflammatory system, also play a role.^6–8^ We hypothesized that unidentified circulating proteins and biological pathways, acting independently or via the hemostasis system, contribute to the development of VTE.

Recently, epidemiological studies leveraging large, relatively agnostic proteomics assays have provided new insights into the etiology and biological mechanisms contributing to chronic diseases including CVD.^9–12^ Similar investigations could provide insights into VTE etiology and would have implications for identifying people at highest risk and developing new preventive methods and treatments. To date, few proteomics studies, limited in either proteomics scale or sample size, have been reported for VTE.^13–16^ Some of those studies had weaknesses such as suboptimal study design (eg, lack of replication^16^ or being cross-sectional^13^). We are not aware of any published large-scale, multi-cohort proteomics studies for incident VTE.

Here, we report on a large proteomics study of VTE in 4 community-based longitudinal cohorts that followed 20 737 participants for a maximum of 10 to 29 years. We used aptamer-based technology by SomaScan to measure plasma levels of ≈5000 to 7000 proteins and examined the prospective relationships between the protein biomarkers and >1300 incident VTEs not attributable to cancer. We further replicated top VTE proteins identified in the 4 cohorts in 783 incident noncancer VTEs among 39 097 participants of UKB-PPP (the UK Biobank Pharma Proteomics Project)^17^ that used Olink assays to measure plasma proteins. Leveraging published data from large genome-wide association studies (GWAS), we also conducted Mendelian randomization (MR) analyses to evaluate possible causal evidence between identified proteins and VTE risk. Last, we conducted a pathway analysis to elucidate biological pathways and molecular networks enriched by the identified proteins.

## Methods

Upon reasonable request, the corresponding author will make the summary statistics, analytical methods, and analytical code available to other researchers, as well as provide information about how to request individual-level participant data.

The institutional review boards for all study cohorts approved the protocols for this project, and all study participants provided informed consent.

### Study Populations and Design

We included 5 population- or community-based cohorts of middle-aged or older participants without a history of VTE who were followed prospectively for VTE occurrence, with the fifth cohort, the UKB study, providing data for replication analysis only. Three cohorts were from the United States: the ARIC study (Atherosclerosis Risk in Communities),^18^ the CHS (Cardiovascular Health Study),^19^ and MESA (the Multi-Ethnic Study of Atherosclerosis).^20^ The fourth and fifth cohorts were from Norway (the HUNT study [Trøndelag Health]^21,22^) and the United Kingdom (UK Biobank study [UKB]^23^), respectively. The design for this multi-cohort study was a prospective investigation of baseline proteomic measurements and incident VTE during follow-up. The Supplemental Material provides details about each cohort. ARIC collected fasting plasma for proteomics assays at visit 2 in 1990 to 1992, when the cohort was ≈48 to 67 years of age, and followed the cohort for VTE through 2019. CHS collected fasting plasma in 1992 and 1993, when the cohort was ≥65 years of age, and followed the cohort for VTE through 2001. MESA collected fasting plasma at exam 1 in 2000 to 2002, when the cohort was 45 to 84 years of age, and followed the cohort for VTE through 2018. HUNT collected nonfasting plasma at the third exam in 2006 to 2008 and followed the cohort for VTE through 2019; we included only HUNT participants ≥45 years of age at baseline. The UKB study collected plasma samples, generally nonfasting, at recruitment between 2006 and 2010, when the cohort was 40 to 69 years of age, and followed the cohort through November 2022 (England), July 2021 (Scotland), or February 2018 (Wales).

ARIC, CHS, and MESA measured proteins in essentially all participants with available baseline plasma (11 994 of the 13 527 participants in ARIC, 3678 of 5265 in CHS, and 5962 of 6814 in MESA). HUNT used a nested case-cohort design, with plasma proteins measured in all incident VTE cases plus a random sample (ie, a subcohort) drawn from the entire HUNT cohort of 50 800 participants who were free of prebaseline VTE, cancer, abdominal aortic aneurysm (AAA), ≥45 years of age at baseline, and had baseline plasma samples. AAA was included in the exclusions because the subcohort was shared between the VTE and an AAA proteomics study (Supplemental Material). HUNT identified 634 incident noncancer VTE cases during follow-up and randomly sampled 1001 participants into the subcohort comparison group (20 participants were in both groups). Of these, 1612 participants (633 VTE cases and 999 subcohort members) had proteomics data returned from SomaLogic.

The proteomics data in the UKB study were generated as part of the UKB-PPP consortium.^17^ We included a majority of samples from the consortium (Supplemental Material).

We meta-analyzed protein–VTE associations from ARIC, CHS, and MESA for discovery and performed replication analyses in HUNT, followed by a second and sequential replication in the UKB study. The sequential replication design in the HUNT and UKB studies is based on consideration of practical factors including availability of the proteomics data in different platforms (ie, SomaScan in ARIC, CHS, MESA, and HUNT versus Olink in the UKB study) and a comparable number of VTE events in HUNT to the total number of VTEs in the 3 discovery cohorts. We also meta-analyzed protein–VTE associations across ARIC, CHS, MESA, and HUNT as a secondary analysis for additional discovery.

### Ascertainment of VTE

All cohorts prospectively followed participants for VTE hospitalizations that occurred after the study exam with proteomic assessment. HUNT and the UKB study also captured outpatient-treated VTE. ARIC, CHS,^24^ and HUNT performed physician reviews of medical records to adjudicate events. MESA^25^ and the UKB study did not conduct medical record reviews and instead used *International Classification of Diseases* (*ICD*) discharge codes to define VTE events. The UKB study also included self-reported VTE diagnoses from baseline nurse interview and death registry codes (Supplemental Material).

We restricted the present study to incident, noncancer-related VTE based on the adjudication in ARIC, CHS, and HUNT or the absence of previous or current hospital *ICD* discharge codes for cancer in MESA and the UKB study. The UKB study also used outpatient records, self-reported diagnoses, and death registry codes to identify cancer cases. We focused on noncancer VTEs to avoid potentially strong confounding effects of cancer on the relationship between plasma proteins and VTE risk.

### Proteomic Measurements and Quality Control (QC) in ARIC, CHS, MESA, and HUNT

SomaLogic (Boulder, CO) performed protein measurements in previously unthawed baseline plasma stored at –80 ºC using the aptamer-based SomaScan V4 5K assay (ARIC and the majority of CHS) with ≈5000 proteins^26^ or the 7K assay (HUNT, MESA, and a small portion of CHS) with the 5000 proteins from the 5K assay plus ≈2000 additional proteins. In this study, we focused on the ≈5000 proteins common to the 5K and 7K panels (Supplemental Material).

#### SomaScan Assays

The SomaScan platform uses SOMAmer reagents (modified single-stranded DNA aptamers) that bind to specific protein epitopes to quantify the concentration of proteins by relative fluorescent units. SomaLogic conducts several steps to standardize and normalize protein measurements^12^ (Supplemental Material).

For all protein SOMAmers included on the 7K panel, the median coefficient of variance (CV)±mean absolute difference across SOMAmers was 4.5±1.5%.^27^ ARIC reported excellent assay reproducibility in a pilot study of SomaScan V3 in 42 ARIC participants: median CV of 5.0 and median intraclass correlation of 0.96.^28^

#### QC Procedures Based on Protein Data

QC filtering of the SomaScan data was conducted using a largely consistent protocol across studies (Table S1). In brief, we log base 2 transformed protein values to correct for skewness of distributions and excluded samples flagged by SomaLogic and outliers based on principal component analysis of all protein values or a proteomic-based sex mismatch analysis (Supplemental Material). We also set to missing any proteins that were >6 SD from the mean and then winsorized protein values that were >5 SD from the mean based on the updated distribution statistics. All 4 studies had excellent quality indices (median CV<7%; Table S1).

### Proteomic Measurements and QC in UKB

Nonfasting blood samples, collected at recruitment and stored at −80 °C, were shipped to the Olink Analyses Service (Uppsala, Sweden) for protein assays using the antibody-based Olink Explore 3072 platform that target 2941 protein analytes.^17^ Olink assays use Proximity Extension Assay technology to measure protein levels.^29^ The UKB-PPP consortium has already applied extensive QC procedures^17^ to protein values, which are represented in normalized protein expression format and log_2_-transformed. We further excluded proteins and samples with >20% missing rates (Supplemental Material).

### Exclusions From Data Analysis

From ARIC, CHS, MESA, and the UKB study, we excluded participants with completely missing proteomics data after the above QC procedures, with a history of VTE before baseline, those taking anticoagulants at baseline (except in the UKB study because of unavailability of the data), those with a self-reported history of cancer at baseline, those whose race was not Black or White (except in MESA and the UKB study) because of small sample size, and those missing the VTE outcome or covariates (details in Table S2 and Supplemental Material for the UKB study). Race/ethnicity in ARIC, CHS, MESA, and the UKB study were self-reported by participants.

The same exclusion criteria were applied to the source cohort in HUNT before identification of VTE cases and subcohort members (see study populations above), except for race and baseline anticoagulant use, which were unavailable. Notably, the population of Trøndelag County (ie, the source population for HUNT), is an ethnically homogenous population of North European ancestry.^21^

### Statistical Analysis

#### Primary VTE Proteomic Analysis

In all 5 cohorts, the study outcome was incident noncancer-related VTE, as defined above. We censored follow-up time for occurrence of cancer-related VTE before a noncancer VTE, loss to follow-up, death, or at the administrative censoring date of each cohort.

For the discovery and replication analyses, the exposure was ≈4955 protein aptamers that passed QC and were common to the 5K and 7K panels in at least 3 of the first 4 studies, or the top VTE proteins we intended to replicate in the UKB study measured by the Olink assays. In each cohort, the hazard ratio (HR) of noncancer VTE per SD increment of each log_2_-transformed aptamer protein or Olink protein was estimated using Cox proportional hazards regression, with adjustment for baseline age, sex, weight, height, estimated glomerular filtration rate (eGFR), and in the US cohorts and the UKB study, additionally for race and field (study) center. The SomaScan platform (5K versus 7K) and shipment batch were additionally adjusted for in CHS and HUNT, respectively. HUNT and the UKB study adjusted for body mass index instead of weight and height. We adjusted for eGFR because kidney function is a VTE risk factor and affects plasma levels of proteins, especially low molecular weight proteins.^30,31^ The Cox regression analyses in HUNT used Barlow’s method^32,33^ for case-cohort designs, weighting each age–sex stratum of the subcohort group by the reciprocals of the sampling fractions and providing robust variance estimation for effect estimates.

##### Meta-Analysis and Replication

ARIC, CHS, and MESA were pooled for discovery analyses, whereas HUNT served as replication to evaluate the reproducibility of significant aptamer protein signals identified from the discovery. HUNT was analyzed as the replication because the number of VTE cases in HUNT was large enough to provide sufficient power to replicate novel aptamer protein signals from the other 3 cohorts. We conducted a fixed-effect inverse variance-weighted meta-analysis to pool protein–VTE associations from the 3 discovery cohorts, followed by a second meta-analysis to pool the association statistics from the 4 cohorts for additional discovery. Cochran’s *Q* test and *I*^2^ statistic were used to evaluate heterogeneity across cohorts. For protein–VTE associations from the meta-analyses, we calculated the false discovery rate (FDR)–adjusted *P* value after adjustment for multiple testing, with *P*<0.05 denoting statistical significance at discovery. Replication testing in HUNT focused on VTE proteins not previously identified in the literature (“new proteins”) from the discovery and used a one-sided Bonferroni corrected *P* value threshold based on the number of new proteins that emerged from the discovery stage: *P*<0.05*2/number of new proteins tested for replication. For proteins identified in the discovery phase that were well-described in the literature (ABO [histo-blood group ABO system transferase], FVIII, VWF, FIX [coagulation factor IX], GDF-15 [growth/differentiation factor 15], and CST3 [cystatin-c]), replication in HUNT was not pursued, but the HRs from HUNT are still presented.

We conducted the second, sequential replication in the UKB study by focusing on the top proteins that were replicated in HUNT at *P*<0.05 and available on the UKB Olink panel, with significant replications based on one-sided Bonferroni corrected *P* value.

##### Secondary Adjustment Analysis

For the significant proteins, in ARIC, CHS, MESA, and HUNT, we additionally adjusted for FVIII using the same Cox regression models as in the primary VTE proteomic analysis; FVIII is a well-known biomarker for VTE, with the most significant association with VTE in our proteomics analysis. We meta-analyzed the adjusted associations across the 4 cohorts and compared the results with those before the FVIII adjustment.

The VTE proteomics analyses were conducted using R 4.2.2 in ARIC, CHS, MESA, and the UKB study, and R 4.1.3 in HUNT; the meta-analysis used METAL.^34^

##### Secondary Analysis to Evaluate Model Fit

To evaluate the improvement of model fit with these identified VTE proteins in addition to established personal risk factors, we calculated concordance index (C-index) from multivariate Cox regressions in 2 models in ARIC, CHS, MESA, and the UKB study: (1) personal risk factors (age, sex, race, height, weight, education [<high school/high school/>high school], smoking status [current/former/never smoker], eGFR, diabetes, and hypertension at baseline); and (2) personal risk factors plus top VTE proteins. In the UKB study, we additionally conducted a formal statistical test of the improvement in C-index using a nonparametric *Z* test implemented in the compareC R package.^35^ The test was not conducted in ARIC, CHS, and MESA, as the top proteins were discovered in those cohorts.

#### MR Analysis

Using summary statistics for proteins and VTE from independent samples, we conducted a 2-sample MR analysis to evaluate the possible causal relationship between the VTE-associated proteins and incident VTE. Instrumental variables (IVs; ie, protein quantitative trait loci [pQTLs]) represented by single nucleotide polymorphisms (SNPs) associated with each identified aptamer protein, came from the published GWAS for SomaScan proteins in 35 559 Icelanders of the deCODE Health study.^36^ We focused on cis-pQTLs within ±1 Mb from the gene boundaries. The following criteria were applied to select IVs (ie, cis-pQTLs) for each protein from deCODE: minor allele frequency >0.01, *P*<5×10^−8^, and being available in the INVENT VTE GWAS data set, which provided SNP–VTE association statistics (n=55 330 VTEs) for the MR analysis.^8^ We then conducted linkage disequilibrium pruning of SNPs based on *R*^2^<0.01 within a ±2 Mb window. Effect estimates from the MR analysis are expressed as odds ratios (ORs) for VTE per SD increase in pQTL-instrumented and rank-inverse normal transformed protein levels. To summarize instrument strength, we report the variance in the proteins explained by the IVs (*R*^2^) and corresponding *F* statistic (Supplemental Material).

Because MR analysis is subject to bias if its assumptions are violated,^37^ we applied the following sensitivity approaches in addition to the primary inverse-variance weighted (IVW) method^38^ to assess the robustness of the results: (1) weighted median,^37^ (2) mode-based estimate,^39^ and (3) MR-Egger methods.^40^ The test of intercept using the MR-Egger method examines presence of horizontal pleiotropy.^40^ Additionally, we used the MR Pleiotropy Residual Sum and Outlier (MR-PRESSO) method to evaluate and adjust for potential horizontal pleiotropy through outlier removal.^41^

Excluding 7 protein aptamers (FVIII, WFDC2 [WAP four-disulfide core domain protein 2], SET [protein SET], COL6A3 [collagen alpha-3(VI) chain:bovine pancreatic trypsin inhibitor/kunitz inhibitor domain, isoform 1], 2 for FIX, and PCDHGA10 [protocadherin gamma-A10]) that did not have suitable IVs (cis-pQTLs) or had <3 IVs, we focused the cis MR analysis on 16 significant proteins from discovery that were replicated in HUNT at *P*<0.05. We considered MR evidence significant based on the following criteria: *P* value < Bonferroni-corrected threshold (*P*<0.05/16=0.0031) in IVW approach plus *P*<0.05 in at least another approach. MR associations with *P* value at 0.0031 to 0.05 from IVW and *P*<0.05 from another approach were considered suggestive. Proteins that showed at least suggestive MR evidence were followed up in a colocalization analysis to strengthen the MR evidence^42^ (Supplemental Material).

##### Sensitivity MR Analysis Based on Replicated IVs (cis-pQTLs)

We conducted a sensitivity MR analysis for 3 new proteins that showed promising evidence from the primary MR analysis by limiting to a subset of IVs that were included in the primary MR analysis and replicated in the meta-analysis of White individuals in ARIC and MESA at *P*<0.05 (Supplemental Material).

All analyses were performed using MendelianRandomization (version 0.7.0)^43^ and TwoSampleMR (version 0.5.6)^44^ packages in R 4.2.2.

#### Pathway Analysis

We used Ingenuity Pathway Analysis (IPA; QIAGEN Inc)^45^ to cluster VTE-associated proteins into pathways and functional groups using FDR-adjusted *P* values <0.05 from the meta-analysis of the 4 cohorts to define VTE-associated proteins (Supplemental Material).

#### Comparison With Protein Measures by Olink Platform

We calculated Spearman correlations for measures of top VTE proteins in log_2_ scale by SomaScan 11K and Olink Explore HT platforms in visit 5 plasma of 102 ARIC participants. Details on the sample characteristics, lab assays, and QCs have been described previously.^46^

## Results

Table 1 presents the final sample size and descriptive characteristics of the 5 cohorts included in the VTE proteomics analysis.

**Table 1.**
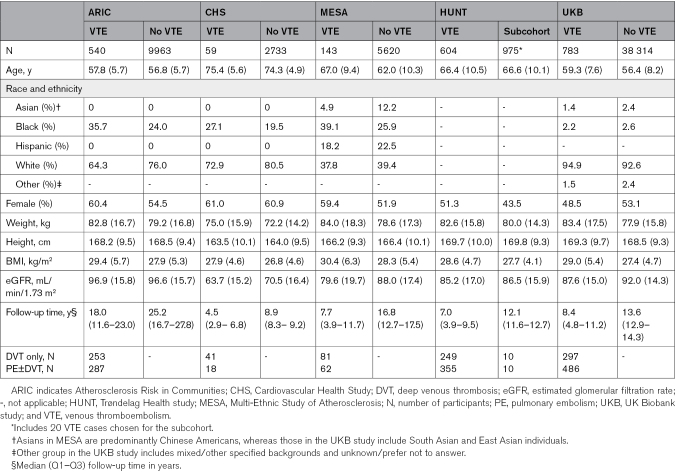
Baseline Characteristics of Study Participants in the Primary VTE Proteomics Analysis (mean [SD] or percentage unless otherwise specified) and Number of Incident Noncancer VTEs

### VTE Proteomic Analysis Findings

**Figure 1.**
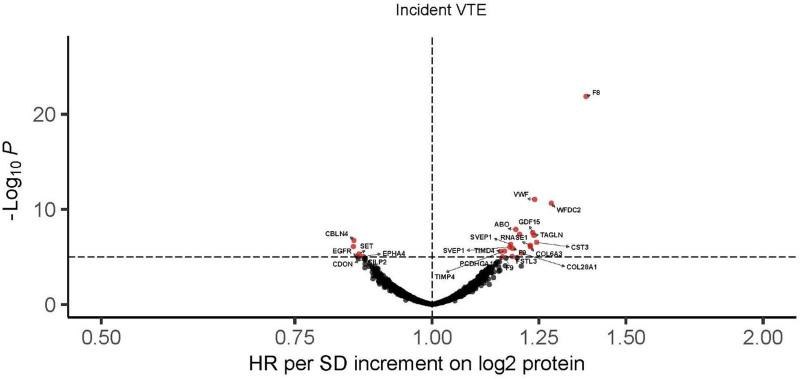
**Volcano plot shows the association of log_2_ protein level with incident noncancer VTE in the meta-analysis of ARIC, CHS, MESA, and HUNT.** The *x*-axis displays the hazard ratio (HR), and the *y*-axis displays the –log_10_ *P* value. The proteins represented by red dots and labeled by gene names are the 23 proteins that exceeded FDR-adjusted *P*<0.05 in the discovery meta-analysis and replicated in HUNT at *P*<0.05 (full names of the proteins are presented in Table 2). The horizontal dash line represents Bonferroni threshold for statistical significance (–log_10_10^-5^). ARIC indicates Atherosclerosis Risk in Communities; CHS, Cardiovascular Health Study; FDR, false discovery rate; HUNT, Trøndelag Health Study; MESA, Multi-Ethnic Study of Atherosclerosis; and VTE, venous thromboembolism.

As shown in Table S3, 67 proteins exceeded an FDR-adjusted *P*<0.05 in the discovery meta-analysis of ARIC, CHS, and MESA, 51 of which have not been previously reported for VTE. Of the 67 proteins, 23 were replicated in HUNT at an uncorrected *P*<0.05 with a concordant direction of association between the discovery and the replication (Figure 1, volcano plot; Table 2; Table S3 and S4). In ARIC, the quality of measures for these 23 proteins was good except for FVIII and VWF (Table S5). Of the 23 proteins replicated in HUNT, 15 are newly described for VTE risk, and the remaining were either established VTE risk biomarkers (FVIII,^3,4^ VWF,^15^ ABO,^16^ and FIX^3,4^; FIX targeted by 2 aptamers) or previously reported in 1 or 2 studies (GDF15,^47^ CST3,^48^ WFDC2/HE4,^16,49^ and COL6A3^16^). Among the 15 new proteins, 3 further exceeded the one-sided Bonferroni-corrected significance threshold in HUNT replication (*P*<0.1/51 new proteins from the discovery stage=1.96×10^-3^): TAGLN (transgelin); SVEP1 (sushi, VWF type A, EGF, and pentraxin domain-containing protein 1); and TIMP4 (metalloproteinase inhibitor 4; Table 2).

**Table 2.**
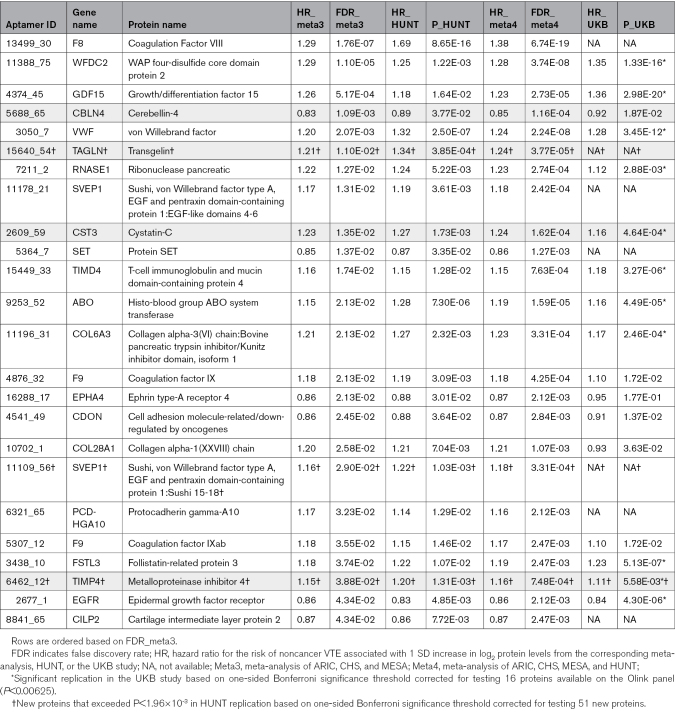
Protein Aptamers that Exceeded FDR-Adjusted *P*<0.05 in the Discovery Meta-Analysis and Replicated in HUNT at *P*<0.05

**Figure 2.**
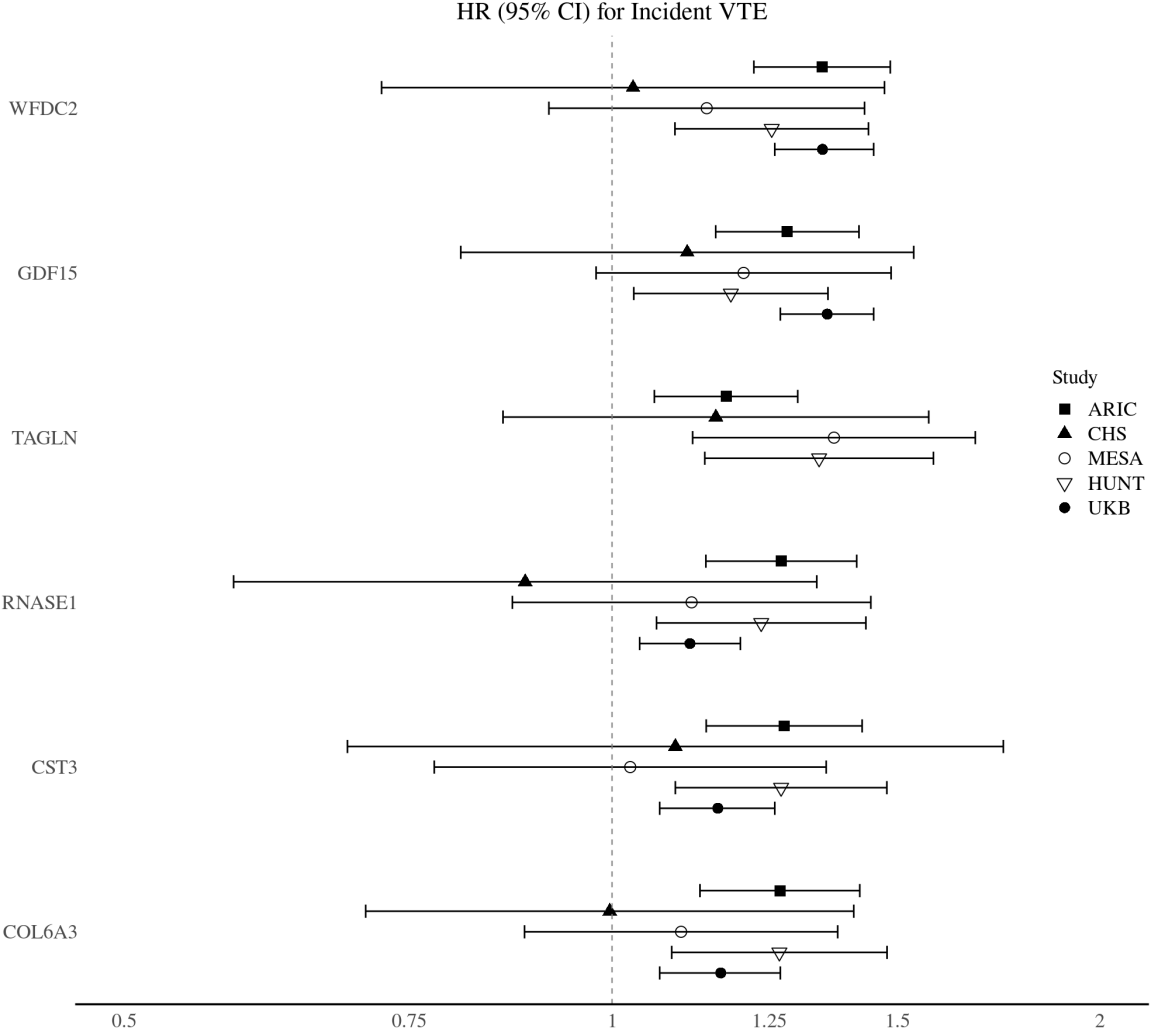
**Forest plot for hazard ratio (HR) and its 95% CI for risk of incident noncancer VTE associated with 1 SD increase in log_2_ protein levels of the top 6 VTE proteins by study.** The top 6 proteins shown are after excluding the well-known VTE proteins FVIII, VWF, ABO, and FIX. As a note, TAGLN is not available in the UKB study. ABO indicates Histo-blood group ABO system transferase; ARIC, Atherosclerosis Risk in Communities; CHS, Cardiovascular Health Study; COL6A3, collagen alpha-3(VI) chain:bovine pancreatic trypsin inhibitor/kunitz inhibitor domain, isoform 1; CST3, cystatin-c; FIX, coagulation factor IX; FVIII, coagulation factor VIII; GDF15, growth/differentiation factor 15; HUNT, Trøndelag Health Study; MESA, Multi-Ethnic Study of Atherosclerosis; RNASE1, ribonuclease pancreatic; TAGLN, transgelin; UKB, UK Biobank study; VTE, venous thromboembolism; VWF, von Willebrand factor; and WFDC2, WAP four-disulfide core domain protein 2.

Among the top 23 VTE proteins replicated in HUNT at <0.05, 16 were available on the UKB Olink panel, and 11 were replicated in the UKB study after Bonferroni correction (*P*<0.0062 in one-sided test), and another 3 had nominal significance at uncorrected *P*<0.05 (Table 2). Figure 2 shows the forest plot for HR (95% CI) of VTE risk associated with the top 6 VTE proteins (excluding the well-known VTE proteins FVIII, VWF, ABO, and FIX) by study based on the effect size from the meta-analysis of ARIC, CHS, MESA, and HUNT.

Table S6 specifically shows the results and QC information for 50 proteins in the clotting pathway that were included on the SomaScan V4 panel. Among them, 15 proteins were significantly associated with VTE at uncorrected *P*<0.05 in the meta-analysis of the 4 cohorts.

There were no strong pairwise correlations among the new proteins or between the new VTE-related proteins and the well-known VTE biomarkers (Table S7). For associations between protein levels and VTE risk, there was no significant heterogeneity in hazard ratios across the cohorts and no violation of the proportional hazards assumption in any individual cohort, based on *P*<1×10^-4^, a moderately stringent threshold that adjusts for multiple testing (Table S3). A second meta-analysis of all 4 cohorts using FDR-adjusted *P*<0.01 for significance identified 9 additional proteins: TREM1 (triggering receptor expressed on myeloid cells 1), BPIFB1 (BPI fold-containing family B member 1), COL13A1 (collagen alpha-1(XIII) chain), IGFBP2 (insulin-like growth factor-binding protein 2), VEGFA (vascular endothelial growth factor A), B4GALT2 (beta-1,4-galactosyltransferase 2), MASP1 (mannan-binding lectin serine protease 1), OGN (mimecan), and SELE (E-selectin) (Table S8); these need evaluation in additional study populations. Meta-analysis results for all 4955 aptamer proteins are shown in Table S9.

For the 23 top proteins, additional adjustment for FVIII abolished the associations for VWF, ABO, and PCDHGA10 (FDR>0.05; Table S10). There were no material changes to the associations for the other 20 proteins, whereas the magnitude of associations decreased modestly for GDF15, RNASE1 (ribonuclease pancreatic), and SVEP1 (3% to 5% change in HR).

Adding the 23 top proteins to established personal risk factors resulted in improvement of model fit based on C-index in ARIC, CHS, and MESA (Table 3). In the UKB study, adding 16 of the 23 top proteins available on the Olink panel statistically significantly improved the C-index beyond the personal risk factors (C-index improvement=0.042; *P*=5.89×10^−9^; Table 3).

**Table 3.**
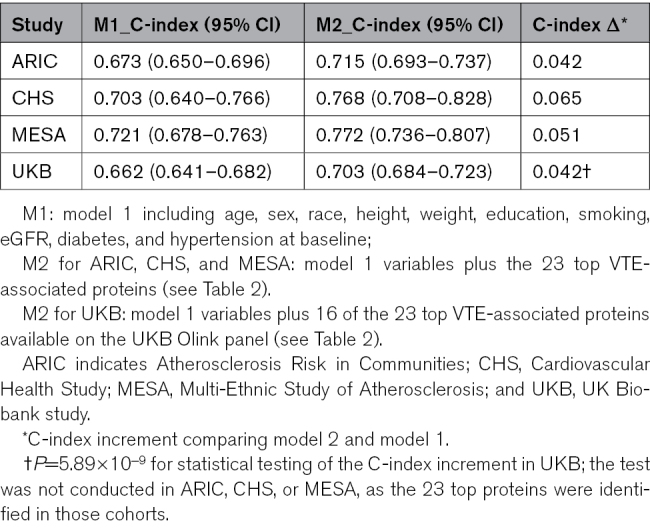
C-Index for Risk Prediction of Noncancer VTE in ARIC, CHS, MESA, and UKB

#### MR Analysis Results

Two-sample MR analysis based on cis-pQTLs was conducted to examine possible causal relationships between identified proteins and VTE. Of the 23 top VTE-associated aptamer proteins, 16 (not including FVIII, WFDC2, SET, COL6A3, 2 for FIX, and PCDHGA10) had suitable pQTL data available in the deCODE SomaScan GWAS database and thus proceeded to the MR analyses. Details on the selected IVs (ie, cis-pQTLs) for the 16 aptamer proteins are in Table S11. Our primary MR analysis confirmed the well-known VTE proteins (VWF and ABO) (Table S12) and identified significant association between a new protein (TIMD4 [T-cell immunoglobulin and mucin domain-containing protein 4]) and VTE after Bonferroni correction (Table 4). There were suggestive associations (*P* at 0.0031 to 0.05 from IVW and *P*<0.05 from another approach) for CST3 and TIMP4 with VTE (Table S12; Table 4). For the 3 proteins, the MR-Egger intercept was not significant, indicating no evidence for pleiotropy (Table 4); no outliers were detected by the MR-PRESSO tests. The colocalization analysis further supported the cis MR evidence between TIMD4 and VTE (posterior probability for colocalization=0.87; Table S13).

**Table 4.**
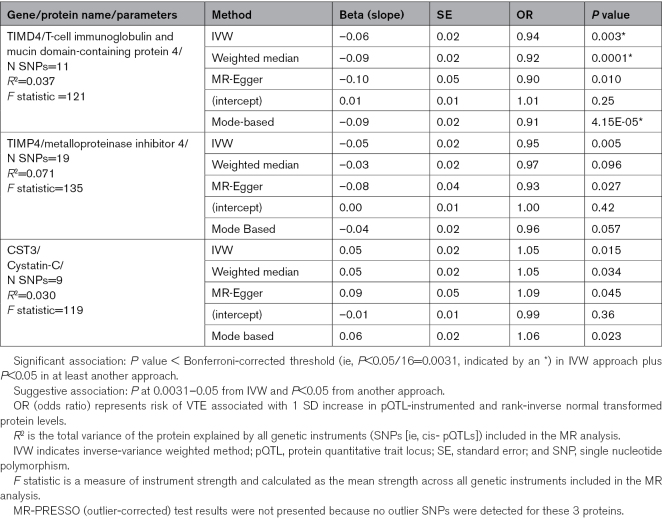
Significant and Suggestive Associations Between the New Proteins and VTE in cis-pQTL Mendelian Randomization (MR) Analyses

For TIMP4 and TIMD4, the direction of association from the MR analyses was opposite to that from the VTE proteomics analysis; OR per SD increase in pQTL-instrumented and rank-inverse normal transformed protein levels was 0.90 to 0.94 for TIMD4 and 0.93 to 0.97 for TIMP4 (Table 4). The directionality of association for CST3 with VTE (OR, 1.05–1.09; Table 4) was consistent to that in the proteomics analysis.

In the sensitivity MR analysis limited to a subset of IVs replicated in the meta-analysis of ARIC and MESA at *P*<0.05 (Table S14), we observed highly consistent findings (Table S15) to those from the primary MR analysis.

### Pathway Analysis Results

**Figure 3.**
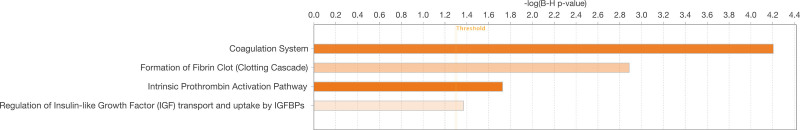
**Biological pathways identified by Ingenuity Pathway Analysis (IPA) analysis meeting the Benjamini–Hochberg (B-H) corrected one-sided *P* value threshold for statistical significance (*P*<0.05).** The top *x*-axis shows the –log (B-H *P* value) after correcting for multiple comparisons. All the pathways displayed are estimated to be activated in influencing the risk of venous thromboembolism (VTE).

Based on FDR-adjusted *P*<0.05 from the meta-analysis of the 4 cohorts, 90 proteins were considered VTE-associated in the pathway analysis. At a one-sided B-H–adjusted *P*<0.05, the pathway analysis provided evidence of involvement of a not–well-known pathway for VTE (regulation of insulin-like growth factor [IGF] transport and uptake by IGFBPs) and confirmed 3 known VTE pathways (Figure 3). IPA-derived *z* scores revealed activation of these 4 pathways (Figure 3). Pathway components are shown in Table S16.

### Comparison With Protein Measures by Olink Platform

Of the 19 significant proteins not previously established in VTE risk, 13 were on the Olink Explore HT panel and measured in plasma samples of 102 ARIC participants. There were strong correlations between SomaScan and Olink measures for 11 of them (Spearman *r*: 0.73–0.92): (CBLN4 [cerebellin-4], COL6A3, RNASE1, TIMD4, TIMP4, WFDC2, EPHA4 [ephrin type-A receptor 4], GDF15, CST3, CDON [cell adhesion molecule-related/down-regulated by oncogenes], and FSTL3 [follistatin-related protein 3]; Table S17).

### Other Validation Evidence for the Binding Specificity of Protein Aptamers

For 18 of the top 25 protein aptamers (target 23 proteins), their binding to the specific VTE proteins have been previously validated by mass spectrometry techniques in the literature: FVIII, WFDC2, CBLN4, VWF, TAGLN, RNASE1, SVEP1 (2 aptamers), CST3, TIMD4, COL6A3, FIX (2 aptamers), COL28A1 (collagen alpha-1(XXVIII) chain), FSTL3, TIMP4, EGFR (epidermal growth factor receptor), and CILP2 (cartilage intermediate layer protein 2);^10,^^50–52^ Table S18.

## Discussion

In this large-scale, prospective, proteomics study of VTE in 1371 incident noncancer VTE events among 20 737 participants followed for a maximum of 10 to 29 years, we identified 23 plasma proteins that were significantly associated with the risk of incident noncancer VTE in the discovery meta-analysis of ARIC, CHS, and MESA and replicated at *P*<0.05 in the HUNT cohort. Of the 23 proteins, 15 were not previously reported for association with VTE in human studies, indicating new proteomics signatures for VTE. Three of the 15 new proteins for VTE reached the more stringent Bonferroni significance threshold in HUNT replication: TAGLN, SVEP1, and TIMP4. In a second and sequential replication in the UKB study based on a different proteomics platform, 11 of the 23 proteins were replicated in the UKB study after Bonferroni correction, and another 3 were replicated at *P*<0.05. MR analyses supported a potentially causal link between TIMD4 and VTE and provided modest support for TIMP4 and CST3. The MR evidence for TIMD4 was further supported by colocalization analysis. Pathway analysis of the broader set of VTE-associated proteins indicated that regulation of IGF transport and uptake by IGFBPs is related to VTE, a pathway not previously established. Taken together, our results provide many new avenues for investigation to identify new etiology of VTE and may ultimately improve its prevention and treatment.

### Biological Relevance of the Top New Proteins in VTE Pathophysiology

#### Experimental Evidence From in Silico Look-Up

In the Mammalian Phenotype Ontology hosted by Mouse Genome Informatics,^53^ we found evidence from animal models supporting the involvement of RNASE1 in abnormal blood coagulation and GDF15 in increased platelet aggregation and increased susceptibility to induced thrombosis (Supplemental Material; Table S19).

#### Other Evidence From the Literature

Transgelin (TAGLN or TAGLN1), also named smooth muscle protein 22-alpha (SM22 or SM22α), is an actin-binding protein, expressed in vascular smooth muscle, and involved in smooth muscle cell contraction and differentiation and cell motility.^54^ Vascular smooth muscle cells play an important role in vascular fibrosis, which contributes to aging and cardiovascular disease.^55^ Animal studies showed that SM22α deficiency promoted vascular fibrosis after arterial injury.^56^ On the other hand, accumulation of SM22α protein accelerates senescence of vascular smooth muscle cells,^57^ which can promote inflammation and activate innate immunity.^58^ Recent human studies reported elevated transgelin in senescent cells,^59^ and an increased risk of incident frailty associated with increased circulating levels of transgelin.^60^ Any role for transgelin in venous endothelial smooth muscle cells is unknown, but it could link venous vessel dysfunction to VTE^61^ via vascular senescence and fibrosis. Interestingly, in an experimental mouse model, transgelin and FSTL3, another top protein in our study, were identified as downstream reactors of the TGFBI pathway, which may play a role in thrombus resolution in chronic thromboembolic pulmonary hypertension.^62^

The alpha-3 chain of type VI collagen (COL6A3) contributes one alpha chain to type VI collagen, which is important in organizing extracellular matrix structure and expressed in nearly all connective tissues including blood vessels.^63^ The interaction of collagens with platelet collagen receptors allows adhesion and activation of platelets.^63^ Platelets from both a collagen VI-null mouse model and patients with collagen VI–related diseases exhibited impaired functions,^64^ accompanied by a moderate bleeding tendency in those patients.^64^

The protein aptamer (11196_31) for COL6A3 associated with VTE risk in our study is believed to target the bovine pancreatic trypsin inhibitor (BPTI)/Kunitz inhibitor domain at the C-terminal of the COL6A3 chain.^65^ The cleavage of the C-terminal Kunitz domain of this protein produces a biologically active fragment, endotrophin.^63^ Endotrophin can act on adipocytes, fibroblasts, macrophages, endothelial cells, and cancer cells to exert pleiotropic effects, including fibrosis, inflammation, angiogenesis, and proliferation of cancer cells.^66^ In prospective human studies, higher circulating levels of endotrophin were associated with a wide range of chronic diseases, including CVD, chronic kidney disease, chronic obstructive pulmonary disease, diabetes, and cancers.^66^ The association between circulating levels of COL6A3 and incident noncancer VTE observed in our study may reflect the effect of endotrophin, but other functions of type VI collagen related to COL6A3, possibly platelet activation and adhesion,^63^ may be involved.

Metalloproteinase inhibitor 4 (TIMP4) belongs to a group of TIMP proteins, which inhibit matrix metalloproteinases (MMPs). Members of the MMP family are involved in the degradation of the extracellular matrix,^67^ which is involved in a variety of biological processes including platelet aggregation and cancer progression.^67^ TIMP4 is the major inhibitor of MMPs inside human platelets and is involved in regulation of platelet aggregation and recruitment.^68^ Human recombinant TIMP4 is able to inhibit platelet aggregation in vitro.^68^ Moreover, TIMP4 and other family members are involved in the regulation of immune function and inflammation,^69^ partially independent of MMP inhibition. For example, TIMP4 was found to promote inflammation and apoptosis in cardiovascular disease.^70^ Studies have shown that immune cells residing in human cardiovascular structures produce TIMP4, which are upregulated in response to inflammation stimuli in vascular tissues.^71^

We observed that TIMD4, also known as TIM-4, had strong evidence for a potentially causal association with VTE risk. TIMD4 is a phosphatidylserine receptor expressed on immune cells to participate in phagocytosis of apoptotic cells and regulation of T-cells.^72^ Recent studies support the role of TIMD4 in various immune responses including allergy, viral infection, autoimmunity, and tumor immunity.^72^ Blood levels of TIMD4 were elevated in sickle cell disease,^73^ which is a risk factor for VTE.^5^ We postulate that the contribution of TIMD4 to VTE risk may be mediated through immune and inflammation pathways.

For TIMP4 and TIMD4, the MR analysis here suggested that lower levels increase VTE risk, whereas the VTE proteomics analysis showed higher plasma levels associated with a greater VTE risk. Similar patterns have been reported, for example for SERPINA3^74^ and angiostatin^75^ with dementia, and for IL6 and soluble IL6R with coronary heart disease (CHD).^76^ MR analysis reflects genetically determined pathways or mechanisms, whereas associations between circulating proteins and VTE reflect a more complex biology determined by genetics, environment, and other drivers of protein levels (eg, compensatory feedback mechanisms). Actually, compensatory feedback mechanisms seemed to explain the discrepancy between the positive association of an *IL6R* variant with IL6 and soluble IL6R levels and negative association of the *IL6R* variant with downstream signaling and CHD risk.^76,^^77^ The negative association between TIMD4 and VTE in our MR analysis is supported by a newly published study in which *TIM-4* (ie, *TIMD4*) knockout in mouse macrophages aggravated DVT progression and inflammation, indicating a protective effect of TIMD4 on DVT.^78^ Likewise, the positive association between plasma levels of TIMD4 and VTE from our Cox regression might be interpreted as a compensatory feedback increase of the protein level in response to the reduced signaling of the TIMD4 pathway because of genetic influences. This compensatory feedback mechanism may also explain the opposite direction of associations between the MR and Cox regression analyses for TIMP4 with VTE. On the other hand, there is no guarantee that all the assumptions for the MR analysis were met. Therefore, the MR findings for these proteins should be interpreted cautiously. Further study is needed to understand these seemingly paradoxical associations.

WFDC2/HE4 belongs to the WFDC domain family, functions broadly as a protease inhibitor, and is expressed in a variety of tissues.^79^ Although HE4 is widely recognized as a biomarker for tumor diagnosis and progression,^80^ elevated HE4 levels have been reported in other conditions, including renal failure, autoimmune disease, viral infections,^79^ idiopathic pulmonary fibrosis,^81^ and dementia.^75^ The findings from our study support the potential usefulness of HE4 as a biomarker for VTE not related to cancer.

Cystatin C (CST3) is a marker of renal function and an extracellular cysteine protease inhibitor abundantly found in body fluids. Through various mechanisms, CST3 plays a role in inflammation, autophagy, bone remodeling, antigen presentation, apoptosis, and inhibition of tumor progression.^82,^^83^ The association of CST3 with VTE in our study is probably not attributable to the confounding effect of impaired kidney function because we adjusted for eGFR in both the discovery and replication stages of the VTE proteomics analysis. In a previous Tromsø study report, CST3 was associated with the risk of VTE in participants with normal kidney function.^48^ Our consistent evidence from multiple MR approaches adds another layer of support for the biological relevance of CST3 to VTE pathogenesis.

Using the same proteomics data and study design with follow-up restricted to 5 years for overall VTE in the HUNT study, Brækkan et al analyzed 294 incident VTE cases, including cancer-related VTEs, and 1066 random subcohort members.^16^ They identified 4 novel proteins (RGS3 [regulator of G-protein signaling 3], PXDN [peroxidasin], COL6A3, and WFDC2) significantly associated with a higher VTE risk after Bonferroni correction and without an established role in VTE pathophysiology. Our study extended the findings for COL6A3 and WFDC2 to noncancer VTE in a longer follow-up. Notably, RGS3 or PXDN was not associated with noncancer VTE in our discovery meta-analysis of ARIC, CHS, and MESA (*P*>0.2). In our HUNT analysis of a longer follow-up, RGS3 was not associated with noncancer VTE (*P*>0.3), and PXDN showed a weaker association (HR, 1.28; *P*=0.001) than that reported in Brækkan et al for all VTE (HR, 1.36; *P*=3×10^-6^).

### Strengths and Limitations

Strengths of the study include a prospective design using large, population-based cohorts with long follow-up, focus on noncancer incident VTE to avoid confounding by cancer, unbiased scanning of numerous protein biomarkers, replication in an independent cohort using a different proteomics platform, and inference of potential causality using MR approaches.

Several limitations of this study warrant mention. First, a single measure of proteins may not characterize long-term levels over the lengthy follow-up. However, moderate correlations were observed for most top proteins across ARIC visits 2 (1990 to 1992), 3 (1993 to 1995), and 5 (2011 to 2013; Table S20). Second, we did not adjust for confounding variables that may have varied over the long follow-up, so residual confounding is possible, but not likely at the MR step. Third, only HUNT and the UKB study included outpatient VTEs, but pilot work suggests that most of the VTE patients in the other 3 studies were hospitalized, so few cases were missed. Misclassifying the few outpatient VTEs as non-VTEs in our discovery stage would bias the protein–VTE associations only if the protein levels were related to hospitalization for VTE. Yet, the HUNT and UKB study replications, which included both hospitalized patients and outpatient VTEs, may provide data to clarify any possible bias. Fourth, VTEs were not validated in MESA or the UKB study, but *ICD* discharge codes are reasonably accurate.^24^ The UKB study also included some self-reported VTE diagnoses from baseline nurse interview and death registry codes, which may not be as accurate as hospital records and thus add noise to VTE diagnoses, reducing statistical power to replicate protein–VTE associations. Fifth, in contrast to the 3 US cohorts that collected fasting samples, HUNT and the UKB study collected nonfasting samples,^9,^^17^ which may have added noise to any fasting status-sensitive protein measures in HUNT and the UKB study and thus reduce statistical power. At last, model fit (C-index) indicated that the top VTE proteins improved the prediction of VTE beyond the established personal risk factors. However, it requires a comprehensive approach, including the use of innovative statistical and machine learning modeling, to yield appropriate prediction models for VTE that can be applied to clinical and preventive practices, which is beyond the scope of this study.

In conclusion, leveraging data from multiple cohorts, we identified new plasma proteins and biological processes related to VTE risk that are outside established VTE pathophysiology, including extracellular matrix regulation, immunity, immune-vascular endothelium interactions, and vascular senescence and fibrosis. Notably, a few of the newly associated proteins (COL6A3 and EPHA4) were considered as drug targets for other conditions (Table S4). Current antithrombotic preventive and treatment therapies for VTE focus on anticoagulants, whereas antiplatelet medications have been investigated but with mixed results.^84,^^85^ Bleeding is a major side effect of antithrombotic treatment.^86^ The new findings here expand understanding of the etiology of VTE and may provide potentially new modifiable targets for intervention to improve risk stratification, prevention, and treatment that might be safer. Future studies are needed to generalize our findings to other populations and to conduct preclinical research necessary to gain better mechanistic insights.

## Article Information

### Acknowledgments

The authors thank the staff and participants of the ARIC study (Atherosclerosis Risk in Communities) for their important contributions; the other investigators, staff, and participants of the MESA study (Multi-Ethnic Study of Atherosclerosis) for their valuable contributions; and the investigators, staff, and participants of the UKB study (UK Biobank) for their valuable contributions. The authors also acknowledge SomaLogic Operating Co, Inc as the provider of the proteomic data measured using the modified aptamer-based SomaScan® Assay. SomaScan®, SOMAmer®, and SomaSignal^TM^ are trademarks of SomaLogic Operating Co., Inc.

### Disclosures

B.M.P. serves on the steering committee of the Yale Open Data Access Project funded by Johnson & Johnson. P.G. serves on a medical advisory board to SomaLogic Inc, for which he accepts no salary, honoraria, or any other financial incentives. For all studies, the content is solely the responsibility of the authors and does not necessarily represent the official views of the National Institutes of Health.

### Author Contributions

W.T. and A.R.F. obtained funding and designed the study. W.T. and W.G. supervised the data analysis. W.T. wrote most of the manuscript. A.L. conducted most of the data analysis, wrote part of the Methods, and led the design of the prediction model analysis in ARIC, CHS, and MESA. T.R.A. contributed to interpretation of CHS data. S.K.B., J.-B.H., and C.J. ascertained VTEs in HUNT. T.H.N. analyzed HUNT data. X.L. analyzed UKB data. P.G. obtained SomaScan data in MESA and contributed to data interpretation. R.C. contributed to the Mendelian randomization analysis, conducted the in silico look up in the Mammalian Phenotype Ontology, and analyzed the lookup data. J.-B.H. adjudicated VTEs in HUNT. R.C.H. conducted the assay validation analysis in ARIC. G.L. contributed to the Mendelian randomization analysis. M.C. and A.R.F. adjudicated VTEs in ARIC and CHS. M.C. provided hematology expertise for data interpretation and contributed to study design. All authors were involved in critical revision of the manuscript for intellectual content. All authors read and approved the final version of the manuscript.

### Supplemental Material

STROBE Cohort Checklist

Supplemental Methods

Supplemental Results

Supplemental Discussion

Tables S1, S2, and S17

Excel Files S3–S16 and S18–S20

References 87–109

## Supplementary Material


