# Supplementary material for: Novel Plasma Proteomic Markers and Risk of Venous Thromboembolism
Source: Circulation. 2026 Feb 16;153(11):810–25. doi: 10.1161/CIRCULATIONAHA.125.074493 (PMC12991346; doi:10.1161/CIRCULATIONAHA.125.074493)
Supplement: Supplementary file 1 [file cir-153-810-s001.pdf]

Novel Plasma Proteomic Markers and Risk of Venous Thromboembolism

Weihong Tang, Aixin Li, Thomas R. Austin, Sigrid K. Brækkan, Therese H. Nøst, Xumin Li, Rajat Deo, Ruth Dubin, Peter Ganz, Weihua Guan, Rui Cao, John-Bjarne Hansen, Kristian Hveem, Ron C. Hoogeveen, Christian Jonasson, Jerome I. Rotter, Kunihiro Matsushita, Guning Liu, James S. Pankow, Nathan Pankratz, Bruce M Psaty, Kent D. Taylor, Florian Thibord, Eric Boerwinkle, Nicholas L. Smith, Mary Cushman, and Aaron R. Folsom

SUPPLEMENTAL MATERIALS

Table of Contents

SUPPLEMENTAL METHODS **4**

Atherosclerosis Risk in Communities Study (ARIC) **4**

Cohort Description and Proteomics Samples 4

Ascertainment of Venous Thromboembolism (VTE) and Covariate Measurements 4

Genotyping 5

Statistical Methods for VTE Proteomics Analysis 6

Cardiovascular Health Study (CHS) **6**

Cohort Description and Proteomics Samples 6

Ascertainment of Venous Thromboembolism and Covariate Measurements 8

Statistical Methods 8

Trøndelag Health study (HUNT) **9**

Cohort Description 9

Ascertainment of Venous Thromboembolism (VTE) and Covariate Measurements 9

Study Design for the Proteomics Study 10

Proteomics Samples 11

Statistical Methods 11

Multi-Ethnic Study of Atherosclerosis (MESA) **12**

Cohort Description and Proteomics Samples 12

Ascertainment of Venous Thromboembolism (VTE) and Covariate Measurements 12

Genotyping 13

Statistical Methods for VTE Proteomics Analysis 14

SomaScan Assays and QC in ARIC, CHS, MESA and HUNT **15**

SomaScan Assays 15

QC Procedures Based on Protein Data 15

Replication Analysis of Top VTE Proteins in the UK Biobank Study **16**

Cohort Description 16

Ascertainment of Venous Thromboembolism (VTE) and Covariate Measurements 17

Proteomics Data by Olink Assays 19

Sample Exclusions 20

Data Analysis 21

Statistics in Mendelian Randomization (MR) Analysis **21**
Colocalization Analysis **22**
Pathway Analysis of VTE Proteins **22**
Sensitivity MR Analysis Based on Replicated IVs **23**
Replication Analysis of IVs in ARIC and MESA 23
In Silico Look-up of Animal Models for Experimental Evidence **24**
SUPPLEMENTAL RESULTS **25**
VTE Proteomic Analysis Findings **25**
Search for Drug Targets Linked to Top VTE-associated Proteins **25**
In Silico Look-up of Animal Models for Experimental Evidence **25**
SUPPLEMENTAL DISCUSSION **26**
SUPPLEMENTAL TABLES **27**
LEGENDS TO SUPPLEMENTAL TABLES IN EXCEL FORMAT **30**

SUPPLEMENTAL METHODS

The institutional review boards for all study cohorts approved the protocols for this project, and all study participants provided informed consent.

Atherosclerosis Risk in Communities Study (ARIC)

Cohort Description and Proteomics Samples

The ARIC investigators initiated a cohort study of 15,792 Black or White participants aged 45-64 years from four US communities in 1987-89 (visit 1).[18] ARIC reexamined 14,348 participants at visit 2 in 1990-92, collected EDTA plasma samples, and saved them at -80 ºC at a central laboratory. In 2021, the ARIC laboratory pulled, aliquoted, and shipped 11,994 remaining visit 2 samples to SomaLogic, Inc., who performed the 5K SomaScan, which included 4955 aptamer targets. For external quality assessment of SomaScan, the ARIC laboratory inserted blind split-sample duplicate plasma specimens for 625 of the 11,994 visit 2 participants; the median split sample reliability coefficient across all aptamers was 0.93. ARIC also obtained SomaScan data for plasma samples collected at visit 3 (1993-95) on 11,563 participants and at visit 5 (2010-11) on 5,237 participants, with median reliabilities of 0.88 at visit 3 and 0.96 at visit 5. The median inter-assay coefficients of variation for proteins were calculated using the Bland-Altman method (CVBA), since proteins levels are measured on a relative scale, with a CVBA of 6% at visit 2, 12% at visit 3, and 7% at visit 5. QC exclusions based on the protein data are described in Table S1.

Ascertainment of Venous Thromboembolism (VTE) and Covariate Measurements

ARIC maintained longitudinal contact with participants to identify all hospitalizations. We identified potential cases of hospitalized VTE from visit 2 through 2019 from ICD discharge codes, and two physicians (ARF and MC) validated VTEs by hospital record review as previously reported.[24] VTE events included deep vein thrombosis (DVT) and pulmonary embolism (PE). Classification as VTE required positive imaging. Cancer-related VTE was defined as VTE within 12 months of active cancer or chemotherapy.

At each visit, ARIC conducted interviews and clinic examinations to collect information on cardiovascular risk factors and conditions including anthropometric measures, smoking, education, history of physician-diagnosed diabetes and other cardiovascular conditions, and medication use. ARIC staff measured weight and height with participants in scrub suits and took three blood pressure measures with a random-zero sphygmomanometer. The average of the last two measurements was included for analysis. Hypertension was defined as systolic blood pressure (SBP) ≥140 mm Hg or diastolic blood pressure(DBP) ≥90 mm Hg or taking antihypertensive medication within the past two weeks. Prevalent diabetes mellitus was defined as a fasting glucose level ≥ 126 mg/dL, nonfasting glucose level ≥ 200 mg/dL, a self-reported physician diagnosis of diabetes, or treatment for diabetes. Medication use was based on medication bottles participants brought to the visit. Information on smoking was obtained by interview and participants were classified as current, former, or never smokers.
Estimated glomerular filtration rate (eGFR) (mL/min/1.73 m^2) at visit 2 was estimated by the Chronic Kidney Disease Epidemiology Collaboration (CKD-EPI) combined creatinine-cystatin C equation.[87] Serum creatinine was measured by a creatinase enzymatic method on a Roche Modular P Chemistry Analyzer (Roche Diagnostics, Indianapolis, Indiana). Serum cystatin C level was measured by a turbidimetric method (Gentian AS, Moss, Norway).

Genotyping

Whole blood genomic DNA samples for nearly the whole cohort were genotyped at the Broad Institute using the Affymetrix Genome-Wide Human SNP array 6.0 (Affymetrix, Santa Clara, CA, USA). Genotypes were called using Birdseed software. QC filtering included exclusion of participants who had a call rate <95% or whose genotype was discordant with known sex or finger-printing genotyping. Detailed information on genotyping and quality control has been detailed previously.[88] ARIC conducted race-specific imputation of variant dosages to the TopMed reference panel.[89] QC procedure prior to imputation included exclusion of individuals who were first-degree relatives, genetic outliers, or whose array genotypes did not match existing genotype data by other platforms. Principal components (PCs) based on the GWAS data were generated using EIGENSTRAT[90] to reflect population substructure or genetic ancestry of ARIC participants.

Statistical Methods for VTE Proteomics Analysis

The study outcome was incident non-cancer related VTE after ARIC visit 2; we censored follow-up time if a participant had cancer related VTE before a non-cancer related VTE. Person-time of follow-up otherwise accumulated from the date of ARIC visit 2 until the date the participant became lost to follow-up, died, or reached the study end date of December 31, 2019.

We ran Cox proportional hazards models to estimate the hazard ratio of VTE per standard deviation (SD) increment of each log base 2 protein. We adjusted for age, race, field center, sex, weight, height, and eGFR. For each significant association (FDR-adjusted p<0.05 in the meta-analysis of ARIC, CHS, and MESA), we verified the aptamer was not appreciably skewed and that the proportional hazard assumption was not violated after adjustment for multiple testing.

Cardiovascular Health Study (CHS)

Cohort Description and Proteomics Samples

In 1989-90 or 1992-93, CHS investigators recruited 5,888 mainly Black and White participants aged 65 or older in four U.S. communities.[19] Baseline measurement of SomaScan proteins used samples from the 1992-3 visit (the 4th visit in the original cohort, enrollment visit in new cohort). All 3188 participants with previously unthawed plasma were sampled. The considerable missing plasma samples in CHS were for some participants who were included in a previous nested case-control study of arterial cardiovascular disease. For quality control purposes, CHS also selected a random sample of 100 participants with paired unthawed plasma from the 1997-98 examination. CHS shipped all samples on dry ice to SomaLogic, Inc. for assays on the 5k SomaScan. The CHS laboratory randomized samples across the plates, except for the 100 paired samples—keeping paired samples on the same plate. Additionally, 490 previously thawed plasma samples from the 1992-3 visit were later selected for measurement using the 7k SomaScan platform.

SomaLogic returned results on 4,985 aptamers for 3288 samples (3188 participants plus 100 repeat measures) and on 7,288 aptamers for 490 samples. The 7K aptamers include the 5K aptamers, so 5K platform could be studied in all participants measured. The remaining aptamers

on the 7K platform were not studied because only 15 VTE cases had data for these aptamers. We excluded the 100 repeated samples, 22 samples with FLAG by SomaLogic, 6 samples with any PC1-10 deviating by >5SD, and 19 outliers in sex mismatch analysis, yielding 3631 samples in the proteomics analysis of VTE with the common set of 4985 aptamers from the 5K and 7K panels. QC exclusions based on the protein data are described in Table S1.

Calibrators included in the SomaScan assays had a median intra-assay coefficient of variation % of 3.4 (10% - 90%: 1.6 – 7.6) in the 5k data and 3.1 (10%-90%: 1.5 – 7.2) in the 7k, and quality control samples had a median inter-assay coefficient of variation % of 4.4 (10% - 90%: 2.6 – 10.1) in the 5k data and 4.3 (10%-90%: 2.6 – 10.1) in the 7k data. Among the 100 participants for whom plasma was sampled from both the 1992-93 and 1997-98 examinations, the median intraclass correlation coefficient across all proteins was 0.66 (IQR, 0.46 – 0.81).

SomaLogic flagged some aptamers for potential quality concerns (735 in the 7k data and 731 in the 5k data). These aptamers remained in the analysis sample, and users are alerted to interpret results with caution. We followed a SomaLogic recommendation to use their scaling factors for the 7K SomaScan when analyzing 5K and 7K data together.

Flowchart of Inclusion of Participants in CHS Proteomics Measures:

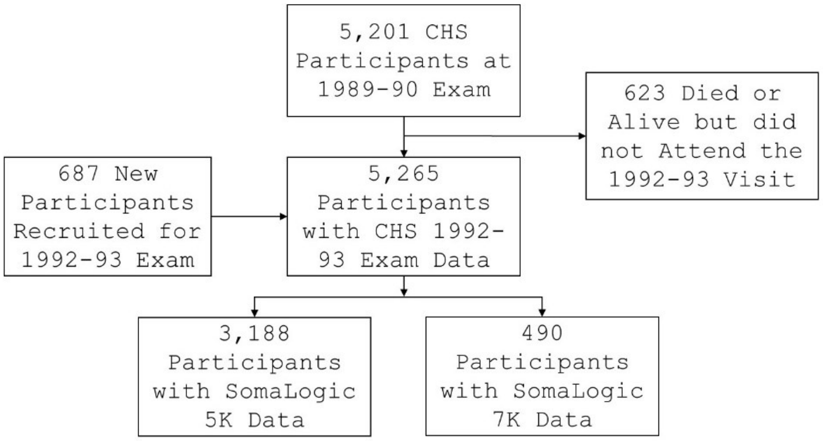

Ascertainment of Venous Thromboembolism and Covariate Measurements

CHS captured and validated VTE from 1992-3 through 2001 using methods identical to ARIC.[24]

In CHS, height, weight, blood pressure, and blood glucose were measured at the 1992-3 visit. Blood pressures were calculated as the average of two seated measures. Participants self-reported their highest level of educational attainment at study baseline and smoking status at the 1992-3 visit. For analysis, smoking status was categorized as (1) Never Smoker, (2) Former Smoker, and (3) Current Smoker, and education was categorized as (1) less than high school, (2) high school graduate, or (3) more than high school. Diabetes was defined as fasting glucose>=126 mg/dL, non-fasting glucose>=200 mg/dL, or use of a glucose-lowering medication. Hypertension was defined as SBP ≥140 mm Hg, DBP ≥90 mm Hg, or current use of antihypertensive medication. The use of antihypertensive medication or glucose-lowering medication was obtained at the 1992-3 visit based on inventory of medication bottles participants brought to the exam. eGFR was measured at the 1992-3 visit using the 2012 CKD-EPI equation[91] from serum creatinine and cystatin C. Serum creatinine was measured using a colorimetric method (Ektachem700; Eastman Kodak, Rochester, NY) and serum cystatin C measured using a BN II nephelometer (N Latex cystatin C; Dade Behring, Munich, Germany).

Statistical Methods

A total of 3,631 CHS participants had data on 4985 aptamers after QC steps described in Table S1. We then successively excluded participants with a history of VTE, taking anticoagulants, with a history of cancer, those whose race was not Black or White, or missing VTE outcome or covariates. This left 2792 participants at risk of VTE, of whom 59 had an incident non-cancer related VTE during follow-up (Table 1).

The study outcome was incident non-cancer related VTE; we censored follow-up time if a participant had cancer related VTE before a non-cancer related VTE. Person-time of follow-up otherwise accumulated from the date of CHS exam in 1992-3 until the date the participant became lost to follow-up, died, or reached the study end date of December 31, 2001.

We ran Cox proportional hazards models to estimate the hazard ratio of VTE per SD increment of each log base 2 protein. We adjusted for age, race, field center, sex, weight, height, eGFR, and platform (5K vs 7K). For each significant association (FDR-adjusted $p<0.05$ in the meta-analysis of ARIC, CHS, and MESA), we verified the aptamer was not appreciably skewed and that the proportional hazard assumption was not violated after adjustment for multiple testing.

Trøndelag Health study (HUNT)

Cohort Description

The Trøndelag Health study (HUNT) is a population-based cohort of inhabitants of Trøndelag county.[21] For the current proteomic-VTE study, we used the third exam (HUNT3), conducted in 2006-2008, for which the investigators invited all inhabitants aged 20 years or older in the former Nord-Trøndelag county to participate. In total, 50,800 individuals took part in HUNT3 (54% of those invited), and of these, 35,065 were 45 years or older. The HUNT investigators followed participants to identify all first-lifetime VTE events through December 31, 2019.

Ascertainment of Venous Thromboembolism (VTE) and Covariate Measurements

HUNT investigators identified all potential VTE cases during HUNT follow-up by searching the hospital discharge diagnosis registry and autopsy registry at the hospitals in Levanger and Namsos, as well as St. Olavs hospital in Trondheim using a broad search with relevant ICD-10 codes for the years 2006-2019. They reviewed the medical record of each potential VTE case and adjudicated and recorded VTE events when signs and symptoms of lower extremity DVT or PE were objectively confirmed by radiological procedures (ultrasound, venography, CTPA, V-Q scan or autopsy) and treatment was initiated (unless contraindications were specified). Cases of concomitantly confirmed DVT and PE were classified as PE. Information on clinical risk factors and potential provoking factors, including cancer, in the three months preceding the event was extracted from the medical records using a standardized form.

Height and weight were measured at the physical examination of HUNT3 baseline with participants wearing light clothing and no shoes, and were used to calculate BMI. eGFR was

estimated at HUNT3 baseline from serum creatinine levels using the 2009 CKD-EPI equation.[92] Serum creatinine was measured by the Jaffe method using alkaline picrate methodology (Abbott, Clinical Chemistry, USA) and then corrected to enzymatic method.

Study Design for the Proteomics Study

We defined prior VTE by ICD-10 codes I26.0, I26.9, I80.0-I80.3, I80.8, I80.9, I82.0- I82.3, I82.8 and I82.9, and cancer by ICD-10 codes C00-C97 (except C44 non-melanoma skin cancer). For this proteomics study, we used a case-cohort design which included all incident VTE cases and a randomly sampled age- and sex-weighted subcohort of individuals from the HUNT3 cohort.
Due to a concomitant project on abdominal aortic aneurysm (AAA), which would use the same subcohort, we incorporated AAA into the case-cohort design.

Prior to sampling the cohort, we excluded HUNT3 participants with a history of VTE (n=622) or cancer (n=1908) before baseline, AAA (n=100) identified by hospital ICD-10 codes during 1999-2006 (before the HUNT3 exam), as well as those without available unthawed plasma (n=1,657), leaving a cohort of 46,513 participants, of whom 31,134 were 45 years or older and served as the source cohort for this HUNT study. Of the 46,513 participants, 911 had an incident VTE during follow-up (n=818 were ages 45+). We randomly sampled the subcohort (n=1,085) with frequency matching on the pooled incident VTE and AAA cases within age (5-year intervals) and sex strata.

In the final case-cohort sample, there were 634 non-cancer VTE cases ≥45 years of age. The subcohort contained 1,001 participants ≥45 years of age, including 20 incident non-cancer VTE cases. Therefore, there was a total of 1,615 unique samples sent for HUNT proteomics assays, of which we received proteomics data for 1,612 participants (633 VTE cases and 999 subcohort members, with 20 VTE cases in both groups).

Flowchart of Inclusion in HUNT Proteomics VTE study:

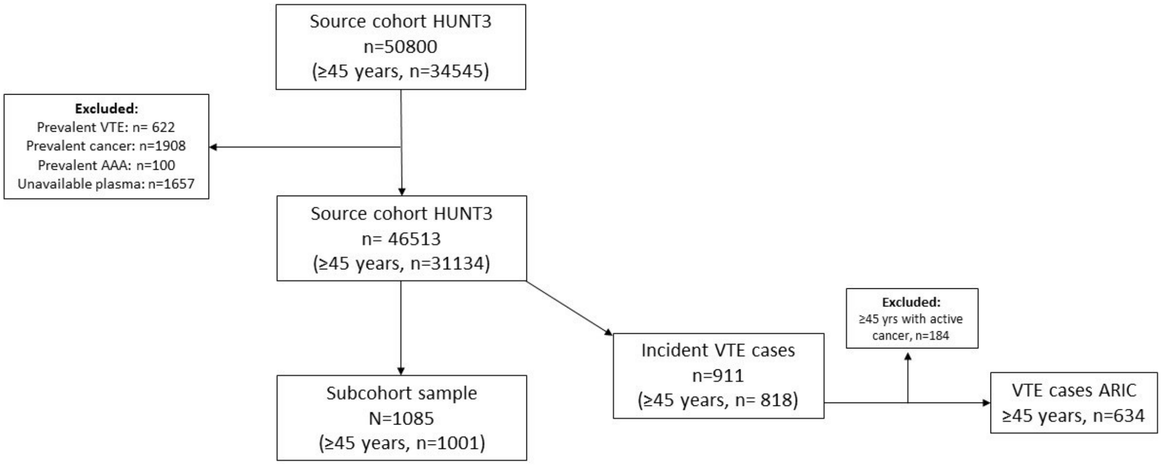

Proteomics Samples

The HUNT3 survey included a physical examination, blood sampling and self-administered questionnaires. Technicians collected blood samples from an antecubital vein, prepared EDTA plasma by centrifugation after one hour respite at room temperature, and froze plasma at -70ºC at the HUNT Biobank in Levanger, Norway. In 2022, HUNT3 investigators retrieved plasma samples for the 634 non-cancer VTE cases and 1,001 subcohort participants and shipped them on dry ice to SomaLogic, Inc. for assays on the 7k SomaScan. SomaLogic returned results for 1,612 participants (633 VTE cases and 999 subcohort members, with 20 VTE cases in both groups). QC exclusions/steps based on the protein data are described in Table S1. After the QC steps, 604 VTE cases and 975 subcohort participants were included in the data analysis (Table 1).

Statistical Methods

Follow-up time was calculated from the date of the study baseline (i.e., inclusion in HUNT3) until the date of an incident VTE, death, loss to follow-up, migration, or end of follow-up, whichever came first. We ran Cox proportional hazards models to estimate the hazard ratio of VTE per SD increment of each log base 2 protein. The Cox regression analyses in HUNT used Barlow's method[32,33] for a case-cohort design, which weighted each age (in 10 years)-sex

stratum of the subcohort group by the reciprocals of the sampling fractions and provided robust variance estimation for effect estimates. The analysis adjusted for baseline age, sex, shipment batch, BMI, and eGFR.

For each significant association (FDR-adjusted $p<0.05$ in the meta-analysis of ARIC, CHS, and MESA), we verified the aptamer was not appreciably skewed and that the proportional hazard assumption was not violated after adjustment for multiple testing.

Multi-Ethnic Study of Atherosclerosis (MESA)

Cohort Description and Proteomics Samples

MESA, initiated in 2000, is a prospective cohort of 6,814 men and women recruited from six field centers across the United States: St. Paul, MN, Baltimore, MD; Chicago, IL; Forsyth County, NC; Los Angeles, CA; and New York, NY.[20] Participants were aged 45–84 years at inclusion and self-identified as Black, Chinese, Hispanic, or White. At baseline, all were free from self-reported cardiovascular disease, active cancer, and other serious medical conditions that might prevent long-term participation in the MESA.

The baseline MESA exam (exam 1) in 2000-2002 collected EDTA plasma and stored it at -80 ^{o}C.

MESA measured proteins in essentially all participants with adequate plasma at exams 1, 4, and 5, of which the baseline exam data was analyzed in this study. For 6,814 participants of MESA baseline exam, SomaLogic returned plasma proteomics data for 5,962 participants. QC exclusions based on the protein data are described in Table S1.

Ascertainment of Venous Thromboembolism (VTE) and Covariate Measurements

MESA conducted VTE surveillance through December 31, 2018. Description of VTE surveillance in MESA can be found elsewhere.[25] Interviewers called participants every 9 to 12 months, and asked about any interim hospital admissions and deaths. MESA obtained copies of hospital discharge diagnoses and recorded International Classification of Diseases (ICD) codes.

VTE was defined by presence, in any position, of the following International Classification of Diseases (ICD)-9 revision codes: 415, 415.x (except 415.0), 451, 451.1x, 451.2, 451.81, 451.9, 453.1, 453.2, 453.4x, 453.5x, 453.8, 453.82, and 453.9 or the following ICD-10 revision codes: I26, I26.0, I26.0x, I26.9, I26.90, I26.92, I26.99, I80.1, I80.1x, I80.2, I80.20, I80.20x, I80.21, I80.21x, I80.22, I80.22x, I80.23, I80.23x, I80.29, I80.29x, I80.3, I80.8, I80.9, I82.1, I82.22, I82.220, I82.221, I82.4, I82.40, I82.40x, I82.41, I82.41x, I82.42, I82.42x, I82.43, I82.43x, I82.44, 82.44x, I82.49, I82.49x, I82.4Y, I82.4Yx, I82.4Z, I82.4Zx, I82.5, I82.50, I82.50x, I82.51, I82.51x, I82.52, I82.52x, I82.53, I82.53x, I82.54, I82.54x, I82.59, I82.59x, I82.5Y, I82.5Yx, I82.5Z, I82.5Zx, I82.9, I82.90 and I82.91. MESA did not capture outpatient VTE, nor did it review medical records to validate hospital VTE discharge codes.

Trained research staff collected information on age, sex, race/ethnicity, education, smoking status, and self-reported medication use through standardized questionnaires at exam 1. They also measured height with a stadiometer and weight with a balance beam scale with participants wearing no shoes and light clothing. BMI was calculated as weight in kilograms divided by height in meters squared (kg/m^2). Participants brought their medications including antihypertensive or diabetic medications to be assessed at the clinic visit by the research staff. Resting SBP and DBP were based on the average of the last 2 of 3 measurements while participants were in a seated position. Hypertension was determined by a SBP $\geq$140 mmHg, DBP $\geq$90 mmHg, or antihypertensive medication use. Participants with a fasting glucose $\geq$126 mg/dL or diabetic medication use were classified as diabetic. A Vitros analyzer (Johnson and Johnson Clinical Diagnostics Inc., Rochester, NY) was used to measure serum creatinine. A BNII nephelometer (Dade Behring Inc., Deerfield, IL) measured serum cystatin C. Both serum creatinine and cystatin C were used to calculate eGFR at exam 1 using the 2021 CKD-EPI equation.[87]

Genotyping

DNA samples from Chinese, Hispanics, and White participants were genotyped using the Affymetrix Genome-Wide Human SNP array 6.0 at Affymetrix Research Services Lab (Santa Clara, California, USA). An additional 1738 samples from Black participants were genotyped at the Broad Institute of Harvard and MIT (Boston, Massachusetts, USA) as part of the CARe

project. Affymetrix performed plate-based genotype calling using Birdseed v2. Sample QC was based on call rates and contrast QC (cQC) statistics. Broad performed similar QC for CARe samples. Additional sample and SNP QCs were carried out, including sample call rate, sample cQC, and sample heterozygosity by ethnicity at the sample level as well as outlier plate checking by call rate, median cQC or heterozygosity at plate level. Plate-based heterozygosity check found no evidence of contamination. Therefore, all plates that passed the other QC metrics were retained. Cryptic sample duplicates based on IBD/IBS were dropped.

MESA excluded monomorphic SNPs across all samples; SNPs with missing rate > 5% or observed heterozygosity > 53% were also excluded.

Genotype imputation was conducted using the TOPMed[89] r2 reference panel. Before imputation, quality control procedure included filtering for a sample call rate above 98%, eliminating duplicate variants, and ensuring a SNP call rate of at least 95%. Variants not meeting Hardy-Weinberg Equilibrium (p-value $<10^{-6}$) were also excluded. For the X chromosome SNPs, male data were handled to mitigate haploid imputation issues by treating all males as females and marking heterozygous X variants as missing.

Statistical Methods for VTE Proteomics Analysis

A total of 5,847 MESA participants with baseline data on 7K aptamers remained after the QC steps described in Table S1. We further excluded participants with a pre-baseline history of VTE, taking anticoagulants, with a history of cancer, or missing VTE outcome or covariates. This left 5763 participants at risk of VTE, of whom 143 had an incident non-cancer related VTE during follow-up. In MESA, the history of VTE prior to exam 1 was unavailable but we excluded a few participants whose VTE diagnosis occurred in the same year as exam 1 baseline. Detailed count on the exclusions is shown in Table S2.

The study outcome was incident non-cancer related VTE after MESA exam 1; we censored follow-up time if a participant had cancer related VTE before a non-cancer related VTE. Person-time of follow-up otherwise accumulated from the date of baseline until the date the participant became lost to follow-up, died, or reached the study end date of December 31, 2018.

We ran Cox proportional hazards models to estimate the hazard ratio of VTE per standard deviation increment of each log base 2 protein. We adjusted for age, race, MESA field center,

sex, weight, height, and eGFR. For each significant association (FDR-adjusted p<0.05 in the meta-analysis of ARIC, CHS, and MESA), we verified the aptamer was not appreciably skewed and that the proportional hazard assumption was not violated after adjustment for multiple testing.

SomaScan Assays and QC in ARIC, CHS, MESA and HUNT

SomaScan Assays

The SomaScan® Platform uses SOMAmer® reagents, modified single-stranded DNA aptamers, to bind to specific protein epitopes to quantify the concentration of proteins by relative fluorescent units. The readout is performed using Agilent hybridization, scan, and feature extraction technology.

Utilizing hybridization normalization control sequences, human calibrator control pooled replicates and quality control pooled replicates, SomaLogic conducts the following five steps to standardize and normalize protein measurements by SomaScan v4 Assay: hybridization normalization, intraplate median signal normalization, plate scaling, calibration, and adaptive normalization to a reference using Adaptive Normalization by Maximum Likelihood (ANML). SomaLogic then conducts additional quality control checks by running replicate controls in the SomaScan Assay alongside clinical samples to quantify the quality of each assay by determining the accuracy of the median replicate signal for each SOMAmer reagent compared to the reference. Details can be found elsewhere.[12,93]

QC Procedures Based on Protein Data

We conducted QC filtering of the SomaScan data for each study using a largely consistent protocol, as detailed in Table S1. We evaluated quality indexes of the protein measures using the coefficient of variation (CV) and variance (log2 scale) based on visit 2 blind duplicates for 514 participants in ARIC, 250 pairs of exam 1 samples in MESA, and SomaScan QC samples in CHS and HUNT; the latter two studies did not have available study duplicate samples. We calculated CV using the Bland-Altman method (CVBA),[94] as protein levels are expressed on a relative scale. We excluded samples flagged by SomaLogic for poor quality and log base 2

transformed protein values to correct for skewness of protein distributions, followed by standardization of protein values to a mean of 0 and a standard deviation of 1. As part of the sample QC procedure, we conducted principal component (PC) analysis of all protein values and excluded outliers who were >5 SD outliers based on any PC1 to PC10. We also conducted sex mismatch analysis by regressing reported sex on nine sex-related proteins suggested by SomaLogic (DEFB104A, MSMB, SPINT3, KLK3, PZP, LEP, CGA.LHB, SHBG, and CGA.FSHB), and excluded samples whose sex was >5SD opposite from the protein predicted sex.

Furthermore, for each participant, we set to missing any individual proteins that were off 6 SD from the mean and then winsorized protein values that were off 5 SD from the mean. All four studies had excellent quality indices (Median CVBA<7%, Table S1).

Replication Analysis of Top VTE Proteins in the UK Biobank Study

Cohort Description

The UK Biobank (UKB) Study is a population-based, prospective study that recruited about 500,000 participants aged 40 to 69 years from the UK between 2006 and 2010 (i.e., baseline).[23] Since its baseline, the UK Biobank Study has collected extensive and in-depth biological and health information from participants, including linkage to electronic health records.

The UK Biobank Pharma Proteomics Project (UKB-PPP) is a precompetitive biopharmaceutical consortium to generate multiplex, large-scale proteomic data. UKB-PPP has performed proteomics profiling in non-fasting plasma samples from about 54,000 UKB participants using the antibody-based Olink Explore 3072 platform that target 2,941 protein analytes.[17] The plasma samples were collected at recruitment and stored at −80 °C. The UKB-PPP samples included: 1) UKB participants randomly at the baseline visit (n= 46,595), 2) individuals selected by the UKB-PPP consortium at baseline (n=6,376), and 3) individuals in a case-control study with COVID-19 repeat-imaging data (n=1,268). Details on sample selection have been described elsewhere.[17] Our project does not include the third type of samples.

Ascertainment of Venous Thromboembolism (VTE) and Covariate Measurements

The diagnoses of VTE and cancer in UK Biobank were primarily based on ICD codes from hospital inpatient, outpatient, and emergency records (i.e., NHS HES data), self-reported diagnoses during the baseline nurse interview, and death registry codes. Below is the list of self-report codes and ICD codes that we used to ascertain VTE events, based on the Cardiovascular Disease Diagnosis Criteria established by the University of Washington's Cardiovascular Health Research Unit.[95]

Self-report codes: 1068 Venous thromboembolic disease
1093 Pulmonary embolism
1094 Deep venous thrombosis (DVT)

ICD-9 codes: 4151* Pulmonary embolism and infarction
4511* Phlebitis and thrombophlebitis of deep veins of lower extremities
4512* Phlebitis and thrombophlebitis of lower extremities, unspecified
4518* Phlebitis and thrombophlebitis of other sites
4519* Phlebitis and thrombophlebitis of unspecified site
4530* Budd-chiari syndrome
4531* Thrombophlebitis migrans
4532* Other venous embolism and thrombosis of inferior vena cava
4534* Acute venous embolism and thrombosis of deep vessels of lower extremity
4538* Acute venous embolism and thrombosis of other specified veins
4539* Other venous embolism and thrombosis of unspecified site
6713* Deep phlebothrombosis antepartum
6714* Deep phlebothrombosis postpartum
6732* Obstetrical blood-clot embolism

ICD-10 code: I26* Pulmonary embolism
I278[a] Other specified pulmonary heart diseases
I801* Phlebitis and thrombophlebitis of femoral vein
I802* Phlebitis and thrombophlebitis of other and unspecified deep vessels of lower extremities
I803* Phlebitis and thrombophlebitis of lower extremities, unspecified

I808* Phlebitis and thrombophlebitis of other sites
I809* Phlebitis and thrombophlebitis of unspecified site
I820* Budd-Chiari syndrome
I821* Thrombophlebitis migrans
I822* Embolism and thrombosis of vena cava and other thoracic veins
I824* Acute embolism and thrombosis of deep veins of lower extremity
I825* Chronic embolism and thrombosis of deep veins of lower extremity
I826* Acute embolism and thrombosis of veins of upper extremity
I827* Chronic embolism and thrombosis of veins of upper extremity
I828* Embolism and thrombosis of other specified veins
I829* Embolism and thrombosis of unspecified vein
I82A* Embolism and thrombosis of axillary vein
I82B* Embolism and thrombosis of subclavian vein
I82C* Embolism and thrombosis of internal jugular vein
O223* Deep phlebothrombosis in pregnancy
O229* Venous complication in pregnancy, unspecified
O871* Deep phlebothrombosis in the puerperium
O882* Obstetric thromboembolism

[a] Code should be I27.82 (Chronic pulmonary embolism) but that precision is not available in UKBB

Codes in first occurrence fields: I26* Pulmonary embolism
I82* Other venous embolism and thrombosis

As a note, the first occurrence fields are curated by UKB and represent the earliest recorded date of a diagnosis across multiple sources, including self-report, primary care, hospital inpatient data (ICD-9 and ICD-10), and death registry. These fields are harmonized and coded more coarsely (e.g., I82*, rather than the full I82.4). In our analysis, we used the earliest available date among all sources (self-report, ICD-9, ICD-10, and first occurrence) to ascertain incident VTE.
We defined cancer-related VTE as a VTE event occurring within 90 days after a cancer diagnosis. Participants with cancer-related VTE were censored at the time of the VTE diagnosis related to cancer, and the outcome of our analysis was restricted to non-cancer VTE.

We excluded participants with a history of VTE or cancer at baseline.

Anthropometric data, physical measurements, and blood samples were obtained by trained study staff. Standing height was measured using a Seca 202 stadiometer. Weight was measured on the Tanita BC-418MA body composition analyzer. Serum creatinine concentrations were quantified from baseline blood samples using isotope dilution mass spectrometry (IDMS)-traceable enzymatic analysis on a Beckman Coulter AU5800. eGFR was calculated using the 2009 CKD-EPI equation[92] with serum creatinine concentrations.

An in-person, self-completed touchscreen questionnaire was administered at UKB assessment centers at baseline. Participants sit at a computer with a touchscreen and answered questions themselves (staff can assist if needed). The following touchscreen questions were used to collect information on education and history of smoking, diabetes, and hypertension.

Smoking: “Do you smoke tobacco now?” “In the past, how often have you smoked tobacco?”. Then a standard algorithm was used in UKB to categorize smoking status into current / former / never smoker.

Diabetes and hypertension: "Has a doctor ever told you that you have diabetes?" and "Ever told had high blood pressure?"

Education: "Which of the following qualifications do you have? Select all that apply"

1 College or University degree
2 A levels/AS levels or equivalent
3 O levels/GCSEs or equivalent
4 CSEs or equivalent
5 NVQ or HND or HNC or equivalent
6 Other professional qualifications e.g.: nursing, teaching
7 None of the above
8 Prefer not to answer

Education status was then categorized into <high school, high school, and > high school for the data analysis.

Proteomics Data by the Olink Assays

The proteomic data analyzed in this study were generated using the Olink® Explore 3072 platform by the UKB-PPP.[17] The UKB-PPP consortium has already applied extensive QC procedures, including exclusion of proteins and samples based on detection limits, call rates, and quality control indices such as the Olink QC flags and CV thresholds.[17] The protein values are represented in Normalized Protein eXpression (NPX) format, which are log2-transformed and normalized measures of relative protein abundance after adjustment for technical variation. For our Cox regression analysis with non-cancer VTE as the outcome, we further standardized the NPX values so that hazard ratios represent the VTE risk per 1 SD increase in protein levels based on standardized log2 NPX values.

Sample Exclusions

After exclusions of samples due to missingness for the protein data, quality filtering based on the protein data, or history of VTE or cancer at baseline, the final study population included 39,097 participants, with 1,047 incident VTE cases, of which 264 were classified as cancer-related and 783 as non-cancer related (please see the flowchart below). The median follow-up time was 13.6 years (IQR 1.4 years), accounting for censoring due to cancer-related VTE.

Sample Exclusion Flowchart

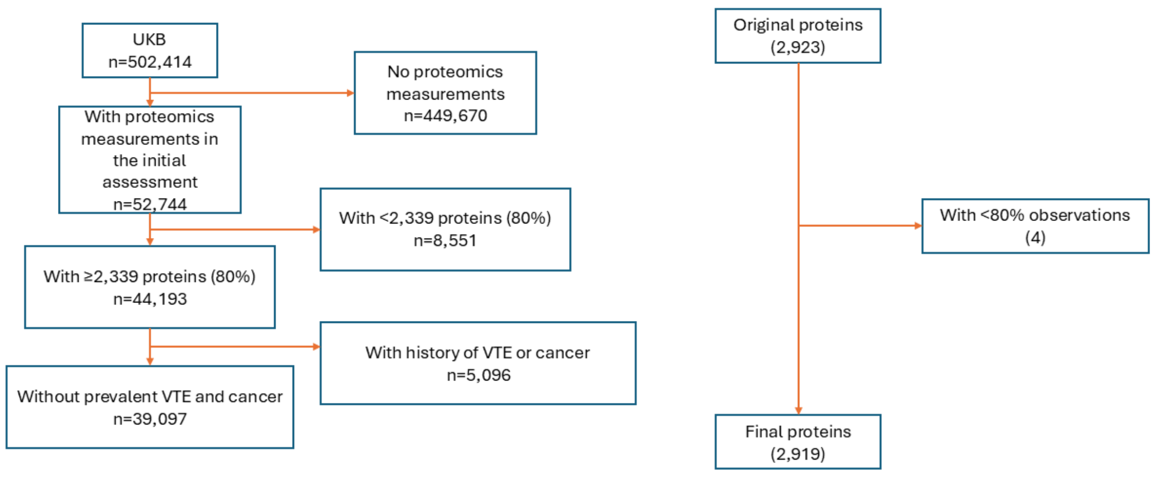

There is no missingness on age, sex, and study center. Missing data for BMI and eGFR were imputed with k-nearest neighbors algorithm (k=5)[96] implemented in the VIM R package. (https://cran.r-project.org/web/packages/VIM/index.html)

Data Analysis

The study outcome was incident non-cancer related VTE after sample collection date for proteomics samples (i.e., analysis baseline); we censored follow-up time if a participant had cancer related VTE before a non-cancer related VTE. Person-time of follow-up otherwise accumulated from the analysis baseline until the date the participant became lost to follow-up, died, or reached the study end date of November 30, 2022 (England), July 31,2021 (Scotland), or February 28, 2018 (Wales).

We conducted Cox proportional hazards regression to estimate the hazard ratio of VTE per one standard deviation increase in protein levels based on standardized log2 NPX values. We adjusted for age, sex, race/ethnicity, study center, BMI, and eGFR at baseline. The study center incorporates information on geolocations. We used one-sided Bonferroni corrected p-value threshold to judge significant replications for protein-VTE associations, i.e., 0.05*2/number of proteins tested in the replication analysis with consistent direction of association to the discovery results for those proteins.

Statistics in Mendelian Randomization (MR) Analysis

R^2 and F Statistic

For each linkage disequilibrium-pruned pQTL instrument j, we calculated the proportion of variance in the protein explained by that SNP using summary statistics from exposure GWAS.[97]

$$\hat{R}_j^2 = \frac{2\beta_{Xj}^2}{2\beta_{Xj}^2 + 2N_X SE_{Xj}^2}$$

where β_{Xj} is the per-allele effect of SNP j on the protein level, SE_{Xj} is its standard error, and N_X is the exposure GWAS sample size from the SomaScan protein GWAS in DECODE.[36] The total variance explained by the K instruments for a protein was obtained by summing across SNPs:[97]

$$R_{total}^2 = \sum_{j=1}^{K} R_j^2$$

We summarized overall instrument strength using the approximate F-statistic:[98,99]

$$F_{total} = \frac{N_X - K - 1}{K} \times \frac{\hat{R}^2_{total}}{1 - \hat{R}^2_{total}}$$

Colocalization Analysis

We conducted colocalization analysis for the three proteins (CST3, TIMD4, and TIMP4) that showed supportive evidence from the cis MR analysis. Colocalization analysis strengthens the interpretation of MR findings by testing whether the genetic association signals for a protein and for VTE are driven by the same underlying causal variant within the protein's cis region.[42]

We used the coloc R package (https://github.com/chr1swallace/coloc), which used a Bayesian colocalization framework to evaluates support for five mutually exclusive hypotheses: H0 (no association with either trait), H1 (association with the protein only), H2 (association with VTE only), H3 (both the protein and VTE are associated but with different causal variants), and H4 (both the protein and VTE are associated with and share the same causal variant). Posterior probabilities were calculated for each hypothesis. We considered posterior probability for H4 > 0.8 as strong evidence of colocalization. We included cis-pQTLs in the analysis, which are defined as SNPs located within ±1 Mb from the gene boundaries. We included SNPs with minor allele frequency > 0.01 that were present in both the deCODE SomaScan protein GWAS and the INVENT VTE GWAS datasets.

Among the three proteins tested, TIMD4 showed strong evidence of colocalization with posterior probability for H4 = 0.875, suggesting a shared causal variant and a direct link to VTE risk. There was no strong evidence of colocalization for the other two proteins with VTE. As a note, a null finding from colocalization analysis does not necessarily refute a positive MR finding.[42]

Pathway Analysis of VTE Proteins

We used Ingenuity Pathway Analysis (IPA, QIAGEN Inc)[45] to cluster VTE-associated proteins into pathways and functional groups. The β estimates (in the form of log expression ratios) and FDR-adjusted p-values from the meta-analysis of the four cohorts were used in the pathway analysis, with the significance threshold set at FDR adjusted p<0.05 for individual proteins.

Protein-VTE associations for the whole set of ~4,955 proteins were uploaded to the IPA platform as a comparison. The Benjamini–Hochberg (B-H) corrected p-value was used to test for enrichment of the identified proteins in specific pathways, compared to the pathway explained by chance alone. Pathways with one sided B-H $p<0.05$ were considered statistically significant. For identified pathways, IPA derived z-scores were used to indicate either activation or inhibition, with the recommended threshold of $z\leq-2$ or $z\geq 2$ used to judge significant directionality.

Sensitivity MR Analysis Based on Replicated IVs

We conducted a sensitivity MR analysis for the three new proteins that showed promising evidence from the primary MR analysis (TIMP4, CST3, and TIMD4). The IVs in the sensitivity analysis were limited to a subset of the IVs (i.e., cis-pQTLs) identified from the deCODE study for the primary MR analysis and replicated in the meta-analysis of ARIC and MESA White participants at $p<0.05$. The sensitivity MR analysis followed the same pipeline as the primary MR analysis and utilized the same summary statistics from the deCODE and INVENT GWAS studies for the replicated IVs.

Replication Analysis of IVs in ARIC and MESA

In the White participants of ARIC and MESA, respectively, we conducted genetic association analyses for each of these three proteins in log2 scale with their corresponding SNPs that were included in the primary MR analysis, using linear regression with adjustment for age, gender, field center, and the first 10 principal components for ancestry. Protein values were after the QC procedure done in the VTE proteomics analysis. A meta-analysis was then conducted using METAL[34] to pool the SNP-protein associations from ARIC and MESA. For each of the three proteins, only SNPs that were replicated at $p<0.05$ in the meta-analysis remained as IVs in the sensitivity MR analysis.

Table S14 presents IVs replicated in the meta-analysis of ARIC and MESA White participants at $p<0.05$. Table S15 presents results of the sensitivity MR analysis.

In Silico Look-up of Animal Models for Experimental Evidence

We further explored the genes encoding the top 23 VTE proteins in animal models. Mammalian Phenotype Ontology, hosted by Mouse Genome Informatics (MGI),[53] is a structured vocabulary primarily consisting of data from mouse and rat models.[100] In the database, we identified 133 VTE-related phenotypes by searching with keywords including coagulation, fibrinolysis, vein, platelet, and thrombosis. Gene set enrichment tests were then carried out using an R package 'enrichR',[101] which implemented Fisher exact tests on the phenotypes and adjusted the p-values by FDR.

SUPPLEMENTAL RESULTS

VTE Proteomic Analysis Findings

As shown in Table S3, 67 proteins exceeded an FDR-adjusted p<0.05 in the discovery meta-analysis of ARIC, CHS, and MESA, and 51 of them have not been previously reported for VTE. The 15 proteins for which circulating levels have been previously associated with VTE are: F8,[3,4] VWF,[15] ABO,[16] WFDC2,[16,49] CST3,[48] COL6A3,[16] F9 (represented by two protein aptamers),[3,4] GDF15,[47] F11,[3,4] TNFRSF1B,[16] SERPINC1,[102] CNTN1,[15] PROC,[102] TNNT2,[103] and CXCL8.[104]

Search for Drug Targets Linked to Top VTE-associated Proteins

We searched for drug targets linked to top VTE-associated proteins in the Therapeutic Target Database (https://db.idrblab.net/ttd/)[105] and IPA database.[45] We noticed that a few of the newly associated or less described VTE proteins (i.e., GDF15, COL6A3, EPHA4, and EGFR) were considered as drug targets for other conditions (Table S4).

In Silico Look-up of Animal Models for Experimental Evidence

As results shown in Table S19, the genes encoding the top VTE proteins are significantly over-represented in six phenotypes (FDR < 0.05). More importantly, genes encoding two VTE proteins that have not been established in VTE risk, RNASE1 and GDF15, were shown to be relevant to the pathogenesis of VTE (Table S19).

SUPPLEMENTAL DISCUSSION

We observed a positive association between ***SVEP1*** and VTE risk. To the best of our knowledge, no previous epidemiological study has demonstrated a potential link between circulating levels of SVEP1 and VTE occurrence. SVEP1, a poorly characterized extracellular matrix glycoprotein, serves as a ligand for integrin α9β1,[106] which plays an important role in mediating cell adhesion, migration, and signaling.[107] SVEP1 also binds to PEAR1, a receptor tyrosine kinase-like protein, to promote platelet activation.[108] Proteomics studies using SomaScan have reported positive associations between circulating SVEP1 and several conditions including coronary artery disease (CAD), type 2 diabetes, heart failure, and cirrhosis.[107] MR analyses supported a causal link for SVEP1 and some of these conditions, including CAD, type 2 diabetes, mean platelet volume, and platelet count.[107,108] Functional studies of SVEP1 in animal models suggested the involvement of SVEP1 in vascular inflammation, including interactions of endothelial cells with leukocytes.[109] Based on these biological data, it is reasonable to hypothesize that the association of SVEP1 with VTE risk observed here may be mediated through mechanisms involving platelet and vascular inflammation. Future mechanistic studies are needed to delineate the mechanisms.

SUPPLEMENTAL TABLES

Table S1. Initial quality control (QC) procedure based on the protein data in the four cohorts.

	ARIC	CHS	MESA (visit 1 data)	HUNT
Protein exclusion in initial QC	non-human, not proteins, missing protein UniProt ID or var*<0.01 or CVBA>50%** (N=325); leaving 4955 aptamers	"deprecated" or non-human (N=253); leaving 4985 aptamers in the 5k panel and 7288 aptamers in the 7k panel	Non-human, not proteins or CVBA† > 50% (N=319); leaving 7277 aptamers	Non-human, not proteins, var<0.01or CVBA‡ > 50% (N=355); Remaining N=7241 aptamers
Samples exclusion in initial QC	19 Samples with FLAG; 53 samples with any PC1-10 deviating by >5 SD; 126 outliers in sex mismatch analysis; leaving 11798 samples	22 samples with FLAG; 6 samples with any PC1-10 deviating by >5SD; 19 outliers in sex mismatch analysis; leaving 3631 in 5k protein analysis	5 samples with FLAG; 69 samples with any PC1-10 deviating by >5SD; 41 outliers in sex mismatch analysis; leaving 5847 samples	16 samples with FLAG; 17 samples with any PC1-10 deviating by > 5SD; zero outliers in sex mismatch analysis; leaving 1579 samples
Median CVBA, %	6.3 (ARIC split QC samples)	4.4 in 5k and 4.3 in 7k (SomaScan QC samples)	5.7 (MESA duplicate samples)	4.7 for the 5k proteins included in the primary analysis (SomaScan QC samples)

*variance in log2 scale
** based on SomaScan QC samples for ARIC visits 2, 3 and 5 assays;
† based on MESA duplicate samples (250 pairs); there are no proteins with CVBA>50% based on SomaScan QC samples;
‡ based on SomaScan QC samples;
FLAG=flagged by SomaLogic for poor quality;
PC= principal component from principal component analysis of the protein data;
CVBA=coefficient of variation (CV) by Bland-Altman method;
Outlier in sex mismatch analysis: reported gender is >5SD opposite from the predicted sex based on 9 sex-related proteins ((DEFB104A, MSMB, SPINT3, KLK3, PZP, LEP, CGA.LHB, SHBG, and CGA.FSHB).

Table S2. Count of sample exclusions in ARIC, CHS, and MESA.

	ARIC	CHS	MESA
No or bad SomaScan data (see Table S1)	2550	47	115
N after above exclusion	11798	3631	5847
VTE prior to sample baseline	223	253	21*
Anticoagulant use at sample baseline	87	64	21
History of cancer at sample baseline	926	457	0
Other race or field site due to small N	36	15	0
Missing outcome or covariates	22	50	42
Final sample in data analysis	10504	2792	5763

Note: sample exclusions in HUNT were done at the sample selection stage for proteomics measures and described in the main manuscript text;
*In MESA, the history of VTE prior to exam 1 was unavailable but we excluded participants whose VTE diagnosis occurred in the same year as exam 1 baseline.

Table S17. Spearman correlation between SomaScan and Olink measures in ARIC samples for selected top VTE proteins.

Aptamer ID	Olink ID	Gene Name	Uniprot ID	Spearman r* (n=102)	CVBA SomaScan (n=115)	CVBA Olink (n=105)
5688_65	OID43457	CBLN4	Q9NTU7	0.86	5.8%	9.3%
11196_31	OID45085	COL6A3	P12111	0.86	4.7%	6.1%
7211_2	OID45406	RNASE1	P07998	0.92	9.1%	8.4%
15449_33	OID45424	TIMD4	Q96H15	0.92	8.6%	8.9%
6462_12	OID45279	TIMP4	Q99727	0.89	5.1%	11.9%
11388_75	OID45018	WFDC2	Q14508	0.83	7.1%	14.3%
16288_17	OID44632	EPHA4	P54764	0.73	4.7%	12.0%
4374_45	OID45131	GDF15	Q99988	0.89	8.6%	6.1%
2609_59	OID45345	CST3	P01034	0.86	5.0%	5.5%
4541_49	OID44530	CDON	Q4KMG0	0.74	4.8%	10.0%
10702_1	OID43551	COL28A1	Q2UY09	-0.04	5.1%	13.7%
3438_10	OID44663	FSTL3	O95633	0.87	5.0%	11.8%
2677_1	OID45115	EGFR	P00533	0.53	4.3%	9.8%
9253_52	OID45299	ABO	P16442	0.88	8.8%	26.7%

*Spearman correlation for protein measures between SomaScan 11K and Olink Explore HT platforms;
CVBA, coefficient of variation by Bland-Altman method; SomaScan: SomaScan 11K assay; Olink, Olink Explore HT assay.

LEGENDS TO SUPPLEMENTAL TABLES IN EXCEL FORMAT

Table	Title
Table S3	Protein aptamer associations with non-cancer VTE in the four cohorts that passed FDR<0.05 in the discovery meta-analysis of ARIC, CHS, and MESA
Table S4	Gene ontology terms, biological locations, protein types, and identified drug targets for top VTE-associated proteins
Table S5	Quality indexes for top VTE protein aptamer measures for ARIC visit 2 SomaScan data
Table S6	Protein aptamer associations with non-cancer VTE for proteins in the clotting pathway from the meta-analysis of ARIC, CHS, MESA, and HUNT
Table S7	Partial Spearman's correlations and p-values among the top VTE proteins and additional known VTE proteins in ARIC visit 2
Table S8	Additional VTE proteins that exceeded FDR-adjusted p<0.01 in the meta-analysis of ARIC, CHS, MESA, and HUNT
Table S9	Full association results between all aptamer proteins and non-cancer VTE in the meta-analysis of ARIC, CHS, MESA, and HUNT
Table S10	Protein VTE associations for the top 24 proteins before and after adjustment for FVIII level in the meta-analysis of ARIC, CHS, MESA, and HUNT
Table S11	SNP-protein associations (IVs or cis-pQTLs) from the deCODE GWAS* included in the primary Mendelian randomization analysis
Table S12	MR results based on cis-pQTLs for the top 16 VTE proteins
Table S13	Colocalization analysis results for proteins that showed promising signals from the cis MR analysis

Table S14 SNP-protein associations (IVs or cis-pQTLs) replicated in the meta-analysis of ARIC and MESA White participants at $p<0.05$ for the sensitivity MR analysis

Table S15 Results of sensitivity MR analysis based on replicated cis-pQTLs in White participants of ARIC and MESA for the 3 new proteins showing promising evidence from the primary MR analysis

Table S16 Significant pathways identified by the IPA analysis for the top VTE proteins

Table S18 External validation of aptamer measurements for the top VTE proteins by two mass spectrometry (MS) techniques

Table S19 Experimental evidence for genes encoding the top VTE proteins from the Mammalian Phenotype Ontology

Table S20 Partial Spearman's correlations between ARIC visits 2, 3, and 5 for the top VTE proteins
